# Influenza-Like Illness Forecasting Using Multisource Data: Comparative Deep Learning Study

**DOI:** 10.2196/91844

**Published:** 2026-08-11

**Authors:** Caixia Dang, Yeqing Tong, Yanquan Mo, Ziqian Zhao, Shanhui Li, Feng Liu, Huiqun Jia, Jingya Zhao, Yuanyong Xu, Hui Chen

**Affiliations:** 1 Chinese PLA Center for Disease Control and Prevention, South Gate, Yard 20, Dongda Street, Fengtai South Road, Fengtai District, Beijing, 100071, China, 86 18149338893; 2 School of Public Health, China Medical University, Shenyang, Liaoning, China; 3 Hubei Center for Disease Control and Prevention, Wuhan, China

**Keywords:** influenza-like illness, multisource data, deep learning, Long Short-Term Memory, Influenza-Like Illness forecasting

## Abstract

**Background:**

Accurate forecasting of influenza-like illness (ILI) is crucial for public health. Integrating novel digital data streams (eg, internet searches and human mobility) with traditional surveillance can improve accuracy, but optimal modeling frameworks are underexplored.

**Objective:**

This study aimed to develop and compare multisource data-driven models for forecasting ILI incidence trends.

**Methods:**

Weekly ILI incidence data and multisource variables for Hubei Province from 2020 to 2023 were collected. Multisource predictors included mean temperature, relative humidity, air quality index, a synthesized Baidu Search Index for influenza-related queries, the Baidu Migration Scale Index for population mobility, and the Oxford Stringency Index (SI) for nonpharmaceutical interventions. Predictive models evaluated were Seasonal Autoregressive Integrated Moving Average (SARIMA), long short-term memory (LSTM), a hybrid convolutional neural network–long short-term memory (CNN-LSTM), Transformer, and random forest. Models were constructed using an 85:15 training-test split and optimized via grid search with 5-fold cross-validation. Performance was assessed using mean absolute error, root-mean-squared error (RMSE), mean absolute percentage error, and coefficient of determination (*R*²).

**Results:**

All models incorporating multisource data substantially outperformed univariate time-series benchmarks. Among univariate models, CNN-LSTM achieved superior performance (*R*²=0.7623) over SARIMA (*R*²=−0.2645). In multisource configurations, the LSTM model demonstrated the strongest predictive capability and feature integration, attaining an optimal *R*² of 0.8350 when combining environmental, mobility (Baidu Migration Scale), and internet search (synthetic Baidu index [SBI]) data. The inclusion of the Oxford SI consistently reduced prediction error across all models, with mean absolute percentage error decreasing by up to 84.87% in the LSTM model. The Baidu Search Index emerged as the most influential single external predictor, notably enhancing model fit. In contrast, model performance varied with feature composition: the Transformer model excelled with full feature sets, while CNN-LSTM performed best primarily with SBI integration. Over a 26-week prospective projection, the optimal LSTM model forecasted a “rapid decline–gradual decline–stabilization” trend, indicating a return to baseline ILI activity, and demonstrated robust external validation performance (*R*^2^=0.7707).

**Conclusions:**

Deep learning models, particularly LSTM, can effectively leverage heterogeneous digital data to improve ILI forecasting. Internet search behavior and policy stringency are critical external predictors. The proposed model demonstrated good external validation performance, and the findings may be applicable to other temperate regions in central China exhibiting similar epidemic characteristics.

## Introduction

Influenza is an acute upper respiratory tract infection caused by the influenza virus [1]. Seasonal influenza epidemics occur annually worldwide, with their intensity and duration varying each year depending on factors such as viral transmission dynamics, population susceptibility, and climatic conditions [2]. During seasonal outbreaks, influenza incidence and mortality rise significantly, imposing sudden and substantial burdens on public health, health care systems, and socioeconomic stability [3]. In China, influenza represents a considerable disease burden and is one of the three leading viral pathogens responsible for acute respiratory infections [4]. Among reported Category C infectious diseases in China, influenza ranks third in both incidence and mortality. The hospitalization rate for influenza in mainland China is 73 per 100,000 population, compared with 35.7 per 100,000 in Hong Kong [5]. During the COVID-19 pandemic, influenza transmission patterns were notably altered. Due to nonpharmaceutical interventions (NPIs), influenza activity in China remained at very low levels from early 2020 until the spring of 2023, when the first postpandemic influenza epidemic season was observed [6,7].

Influenza primarily spreads via respiratory droplets and aerosols, and its transmission dynamics are influenced by a complex interplay of environmental, social, and virological factors. Substantial evidence indicates that environmental conditions such as temperature, humidity, and air quality significantly affect influenza virus transmission [8,9]. Additionally, social determinants—including population mobility, urbanization, and vaccination coverage—also play important roles in shaping its spread. In regions with similar climates and demographic structures, influenza epidemic intensity often varies considerably, with population mobility showing a positive correlation with outbreak magnitude [10]. Moreover, increased urban population density has been suggested as a potential contributor to higher peak prevalence and accelerated transmission during influenza pandemics [11]. Furthermore, public health interventions implemented by governments exert a notable regulatory effect on influenza activity; NPIs in particular have demonstrated substantial effectiveness in suppressing the transmission of influenza and other respiratory infections [7]. Incorporating policy intervention intensity as a covariate in influenza forecasting models is therefore of considerable importance for enhancing model adaptability and predictive accuracy across varying prevention and control scenarios.

Traditional influenza surveillance systems rely primarily on influenza-like illness (ILI) case reports from sentinel hospitals and laboratory-confirmed virological data. This passive, health care–based surveillance model forms the foundation of disease monitoring, providing essential data for analyzing epidemic trends and characterizing viral features. However, a notable time lag exists between patient consultation, sample collection, and data reporting, which limits the timeliness required for early warning. Moreover, due to constraints in surveillance network coverage and health care resource distribution, mild cases and individuals relying on self-medication often do not seek medical care, leading to underestimation of the true incidence [12]. In recent years, the rapid advancement of internet technologies and widespread adoption of smart devices have transformed how people access health information, while also opening new data dimensions for infectious disease surveillance research [13]. In China, Baidu—the dominant Chinese search engine with a market share exceeding 70%—provides search index data that offer broad population coverage and high timeliness. These data have been extensively applied in forecasting the incidence trends of infectious diseases [13-15]. Similarly, population mobility data offer novel perspectives on the spatiotemporal spread of influenza. The Baidu Migration Big Data platform, leveraging mobile positioning and transportation data, enables real-time tracking of the scale and direction of intercity and interregional population movements, thereby supplying high-resolution human mobility information for assessing cross-regional transmission risks of infectious diseases [16]. Social media data and other big data platforms are also being increasingly explored for influenza sentiment monitoring and incidence trend prediction [17,18]. The integration of such diversified internet-based big data with traditional surveillance sources is promoting a shift in influenza monitoring from “passive surveillance” toward “active early warning,” offering unprecedented data foundations and technical potential for constructing a comprehensive, multilayered influenza surveillance and early-warning system.

The development of influenza forecasting models has evolved from traditional statistical methods to machine learning and further to deep learning approaches, with continuous improvements in model complexity and predictive capability. Existing research has predominantly focused on single data sources, while systematic integration of multisource heterogeneous data—such as meteorological and air quality indicators, internet search trends, population mobility, and policy interventions—remains relatively scarce. Consequently, the synergistic predictive value of multisource data has yet to be fully explored. Moreover, systematic comparisons among traditional statistical models, machine learning models, and deep learning models are limited. Variations in datasets, evaluation metrics, and experimental designs across different studies hinder reliable guidance for model selection in practical applications. Furthermore, the adaptability and robustness of existing models under varying epidemic scenarios require further validation, especially in the context of significantly altered influenza transmission patterns following the COVID-19 pandemic. It remains unclear whether current models can effectively adapt to such nonconventional epidemic scenarios. To address these gaps, this study uses ILI surveillance data from the Hubei Provincial Center for Disease Control and Prevention, integrated with multidimensional variables including meteorological data, air quality indices, Baidu search indices, Baidu migration indices, and the Oxford Stringency Index (SI). A multisource data fusion–based influenza forecasting framework is constructed. Five representative forecasting models—SARIMA, random forest (RF), long short-term memory (LSTM), convolutional neural network–long short-term memory (CNN-LSTM), and Transformer—are developed and systematically compared to evaluate their performance in prediction tasks. The optimal predictor combination and best-performing model are selected to forecast future influenza trends in Hubei Province. This study aims to clarify the extent to which multisource data enhance influenza forecasting accuracy, identify key features for trend prediction, and improve the timeliness and accuracy of influenza early warning models. The findings are expected to provide scientific evidence and technical support for optimizing influenza surveillance and early warning systems and for informing public health emergency decision-making.

## Methods

### Data Sources

Weekly ILI data for Hubei Province and its prefecture-level cities from January 2020 to December 2023 were obtained from the Hubei Provincial Center for Disease Control and Prevention. Meteorological and air quality data for Hubei Province covering 2020‐2023, including mean temperature, relative humidity, air quality index, and mean dew point, were acquired from the National Science Data Center platform.

To incorporate the Baidu Search Index (BSI), a literature search was conducted on databases such as PubMed and CNKI to identify studies related to ILI. Frequently used keywords in published papers were summarized, resulting in an initial list of 53 candidate keywords potentially associated with influenza epidemic trends (Table S1 in Multimedia Appendix 1). These keywords included terms related to early ILI symptoms, medications, treatments, and other closely associated phrases, such as “fever,” “body temperature,” and “cough.” The initially screened keywords were subsequently excluded and refiltered (Multimedia Appendix 1). Nine keywords showing significant correlation with influenza activity were finally selected (Table S2 in Multimedia Appendix 1). To avoid multicollinearity, these keywords were aggregated into a synthesized Baidu Index as an internet-based surveillance factor, with higher weights assigned to keywords exhibiting stronger correlations [14] (Multimedia Appendix 1). The composite index was calculated using the following formula:


(1) $${\text{M}}^{\text{W}}\text{(}\text{t}\text{)=}\sum_{\text{i}\text{=1}}^{\text{7}}\frac{{\text{C}}_{\text{i}}}{\sum_{\text{j}\text{=1}}^{\text{7}}{\text{C}}_{\text{j}}}{\text{M}}_{\text{i}}$$

In Formula (1), $\text{\textbackslash{} }{\text{M}}^{\text{W}}\text{(}\text{t}\text{)}$ represents the synthesized Baidu Index, ${\text{M}}_{\text{i}}$ denotes the Baidu Index of the *i-th* keyword, ${\text{C}}_{\text{i}}$ is the maximum correlation between the Baidu Index of the *i-th* keyword and influenza cases.

To dynamically capture weekly variations in population mobility, we selected the Baidu Migration Scale (BMS) Index as a real-time proxy. Derived from the Baidu Map Qianxi platform using location-service data [19], the BMS provides continuous, weekly insights into regional population aggregation and movement directions. For this study, Hubei Province’s immigration and emigration indices from January 2020 to December 2023 were used to measure population movement.

NPIs and policy measures were characterized using the Oxford COVID-19 Government Response Tracker (OxCGRT), an evaluation framework developed by the Blavatnik School of Government research team to systematically track government policies worldwide during the COVID-19 pandemic [20]. To quantify government response intensity, we used the 2020‐2023 Oxford SI for Hubei Province. Given its phased nature—dropping to 0 after China’s COVID-19 policy shift on January 8, 2023—the SI was incorporated as a dynamic variable. When SI>0, it acts as an active feature; when SI=0 (throughout 2023), its input remains 0, effectively ignoring its contribution. Processing details are in Multimedia Appendix 1.

### Data Preprocessing

To rigorously prevent data leakage, the 209-week internal dataset (2020‐2023) was chronologically split prior to any sliding window processing or standardization. Based on an 85:15 ratio, the data were divided into a training set comprising the first 177 weeks (wks 1‐177) and a testing set comprising the remaining 32 weeks (wks 178‐209). Subsequently, a 6-week sliding window was applied independently to each dataset to construct the supervised learning sequences. Because the prediction for a given week t requires features from the preceding weeks *t*-6 to *t*-1 , this independent sliding mechanism inherently consumes the first 6 weeks of each split. Consequently, this process yielded 171 training sequences (dimension: 171×6×6, generating predictions from wk 7 to 177) and 26 testing sequences (dimension: 26×6×6, generating predictions from wk 184 to 209). For normalization, *z* score standardization was applied, with parameters calculated exclusively from the training set to scale the testing data. Finally, K-fold cross-validation was strictly confined to the training set, ensuring the testing set remained completely unseen during model tuning. An additional 26-week dataset from 2024 was later used strictly as an external validation set to evaluate prospective forecasting capability.

### Research Methods

#### Predictor Selection

Spearman rank correlation analysis and generalized additive models were used to assess the correlations and exposure–response relationships between ILI incidence and potential influencing factors [21]. These factors included meteorological and air quality variables, BMS Index, the BSI, and the Oxford SI. Indicators that demonstrated statistically significant associations with ILI incidence and clear public health relevance were prioritized for inclusion in subsequent predictive models (Figures S2-S3 in Multimedia Appendix 1).

#### Predictive Models

##### SARIMA Model

The Seasonal Autoregressive Integrated Moving Average (SARIMA) model is specifically designed for time series data exhibiting pronounced seasonal fluctuations. It captures simultaneously the nonstationarity, short-term dependencies, and stable seasonal periodic patterns in the series [22]. In this study, optimal model configurations were identified through a grid search and evaluated based on the lowest Akaike information criterion (AIC) and Bayesian information criterion (BIC).

##### LSTM Model

The LSTM model is an improved neural network architecture based on recurrent neural networks (RNNs) [23]. While sharing a similar structure with RNN, LSTM incorporates enhanced memory units [24]. LSTM stores long-term information through a memory cell (cell state) and uses 3 “gating mechanisms” to control information updating, forgetting, and output, thereby effectively capturing long-term dependencies in time series [25].

##### CNN-LSTM Model

The CNN-LSTM deep neural network model is a hybrid architecture that combines convolutional neural networks (CNNs) with LSTM networks. The CNN-LSTM model constructs a spatiotemporal sequence prediction framework through spatial feature extraction by the CNN module and temporal dependency modeling by the LSTM module [26]. In terms of model architecture design, the entire network comprises four core components: an input layer, a CNN feature extraction module, an LSTM temporal modeling module, and an output layer [27].

##### Transformer Model

The Transformer model was originally proposed by Vaswani et al [28]. The Transformer model relies entirely on a self-attention mechanism, combining sequential data processing capabilities with parallel computation advantages. The model dynamically weights input nodes to compute correlations across different features, efficiently processing the multisource input data with dimensions ranging from 2 to 6 used in this study.

##### RF Model

RF is a machine learning algorithm based on ensemble learning principles, which enhances model performance by constructing multiple decision trees and aggregating their prediction results [29,30]. By using the Bootstrap Aggregating (Bagging) strategy alongside random feature subset selection, RF effectively prevents overfitting and efficiently handles complex nonlinear relationships.

### Model Construction and Validation

In this study, the SARIMA model was built using the Python statsmodels library, while deep learning and RF models were developed using the PyTorch framework and scikit-learn library. For the SARIMA model, optimal configurations were identified through grid search and evaluated based on the lowest AIC and BIC values. For deep learning and machine learning models, hyperparameter tuning was conducted using a 5-fold cross-validation coupled with a grid search strategy to identify the configuration yielding the minimum average loss (mean squared error [MSE]) [26]. To mitigate overfitting in neural networks, a Dropout mechanism (*P*=.2) was incorporated. During model training, the Adaptive Moment Estimation (Adam) algorithm was used as the optimizer to ensure stable convergence to optimal values [31]. Detailed validation procedures, hyperparameter selection processes, and residual diagnostics are provided in Multimedia Appendix 1.

### Evaluation Metrics

Following model construction and validation, the test set was used for retrospective prediction. Overall model performance was evaluated using mean absolute error (MAE), root-mean-squared error (RMSE), mean absolute percentage error (MAPE), coefficient of determination (*R*^2^), and MSE. Furthermore, to assess the robustness of the models across different forecast horizons, specific evaluations were conducted for short-term (8 wks), medium-term (12 wks), and long-term (26 wks) predictions. These analyses were performed on the same test set by extracting and comparing the predicted values of the final 8-, 12-, and 26-time steps against the corresponding ground truth data. For each specific forecast horizon, performance was comprehensively evaluated using *R*^2^, RMSE, MAE, and MAPE. Specifically, *R*^2^ reflects the overall goodness-of-fit, while MAPE indicates the model’s capacity for relative error control; together, these 2 metrics serve as the primary dimensions for evaluating prediction robustness over varying time lengths.

### Ethical Consideration

**Figure 1.**
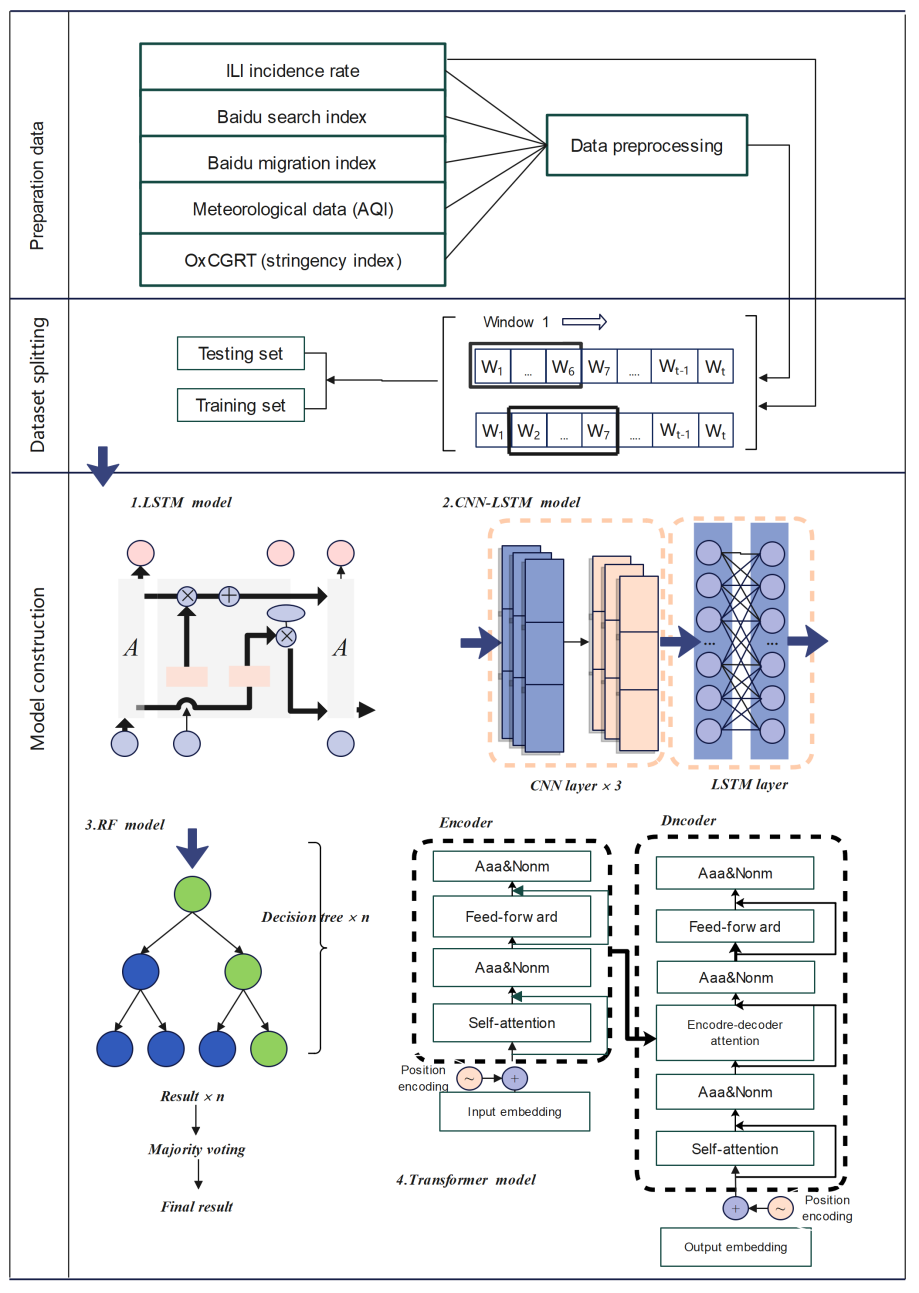
Study design flowchart. AQI: air quality index; CNN: convolutional neural network; CNN-LSTM: convolutional neural network–long short-term memory; ILI: influenza-like illness; LSTM: long short-term memory; OxCGRT: Oxford COVID-19 Government Response Tracker; RF: random forest.

The ILI surveillance data used in this study were provided by the Hubei Provincial Center for Disease Control and Prevention. These data are routine, aggregated weekly statistics from the statutory infectious disease surveillance system and are fully deidentified, containing no personally identifiable information. Because this study relied exclusively on aggregated data and did not involve the collection of individual human participant data, formal institutional review board ethical approval was waived. Furthermore, all meteorological data and internet-derived indices (BSI, Baidu Migration Index, and Oxford SI) were sourced from publicly accessible platforms; these are nonpersonal datasets that do not implicate personal privacy or confidentiality. Finally, as this research did not involve the direct participation of human participants, informed consent procedures were not applicable, and no participant compensation was provided (Figure 1).

## Results

### Overview of ILI Incidence

From January 2020 to December 2023, ILI in Hubei Province predominantly occurred during the winter-spring transition, with reported cases peaking each year in December and January. The first epidemic peak was observed in early 2020, after which incidence declined rapidly and remained at low levels from February 2020 through the end of 2021. In early 2022, ILI incidence began to rise again, followed by a distinct second peak in July 2022, before decreasing once more. After the relaxation of COVID-19 containment measures in 2023, ILI incidence increased sharply, with the highest peak during the observation period occurring in February 2023-March 2023, when the weekly incidence surged to 143.97 per 100,000 population. Another epidemic peak reappeared in the winter of 2023, reaching a maximum weekly incidence of 106.71 per 100,000. Given the substantial fluctuations in ILI incidence associated with NPIs during the pandemic, a logarithmic transformation was applied to the incidence data in subsequent deep learning modeling. This preprocessing strategy helped mitigate the influence of extreme values on model training and enhanced model stability and generalizability (Figure S1 in Multimedia Appendix 1).

### Univariate Time-Series Forecasting of ILI

#### Model Architecture and Hyperparameter Tuning

##### SARIMA

Consistent with previous studies, the ILI incidence series exhibited distinct seasonal patterns. The Augmented Dickey-Fuller test applied to the training dataset indicated nonstationarity in the original series (*P*>.05). After first‑order differencing, stationarity was significantly improved, with an Augmented Dickey-Fuller test statistic of –6.1779 (*P*<.001), confirming that the series had been transformed into a stationary time series. The initial selection of SARIMA parameters was based on the autocorrelation function and partial autocorrelation function plots of the differenced stationary series. The autocorrelation function displayed a slow decay, maintaining significant positive correlations at lags 1-4, while the partial autocorrelation function showed a rapid cutoff after lag 2, indicating a clear trailing 1 pattern. A grid‑search approach was used to compare the AIC and BIC values across different parameter combinations. The model achieved optimal performance with an AIC of –158.13 and a BIC of –144.33, leading to the selection of SARIMA (2,1,2) (1,1,0) as the final model. Diagnostic analysis revealed that the residual series exhibited white‑noise characteristics across various lag orders, with Ljung-Box test results yielding *P*>.05, indicating that the model adequately captured the underlying information in the series and demonstrated satisfactory goodness of fit (Figures S4-S5 in Multimedia Appendix 1)*.*

##### LSTM, CNN-LSTM, and RF

**Figure 2.**
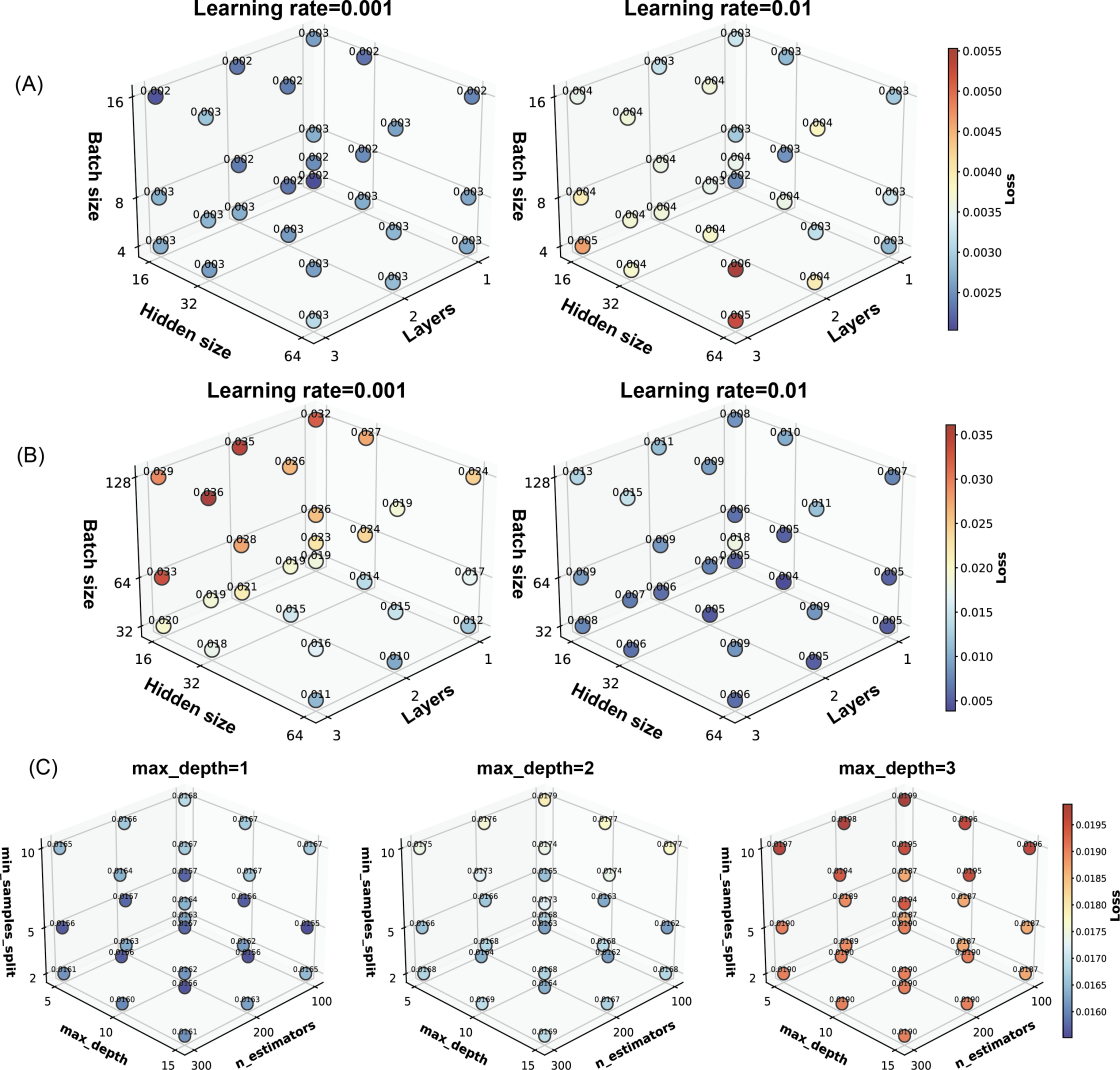
Hyperparameter tuning results for the convolutional neural network–long short-term memory, long short-term memory, and random forest models. (A) convolutional neural network–long short-term memory, (B) long short-term memory, and (C) random forest.

The model development process comprised three stages: model definition, training, and validation. During the definition stage, hyperparameter optimization was performed using grid search combined with 5-fold cross-validation. The hyperparameter search space for the LSTM model included number of LSTM layers (1, 2, 3), hidden units (32, 64, 128), batch size (32, 64, 128), and learning rate (0.001, 0.01). For the CNN-LSTM model, the search space covered: number of LSTM layers (1, 2, 3), hidden units (16, 32, 64), batch size (4, 8, 16), and learning rate (0.001, 0.01), while the number of CNN channels was fixed at 64. For the RF model, the following hyperparameters were explored: number of trees (100, 200, 300), maximum tree depth (5, 10, 15, none), minimum samples required to split an internal node (2, 5, 10), and minimum samples required at a leaf node (1, 2, 4). Grid search was conducted using 5-fold cross-validation with MSE as the evaluation metric to identify the optimal hyperparameter combination. The final selected configurations were as follows: LSTM: LSTM layer: 1, hidden units: 32, batch size: 32, learning rate: 0.01 (Figure 2A); CNN LSTM: LSTM layer: 1, hidden units: 16, batch size: 4, learning rate: 0.001, with CNN channels fixed at 64 (Figure 2B); RF: 200 trees, maximum depth: 10, minimum samples to split: 5, minimum samples at leaf: 1 (Figure 2C). During the training phase, backpropagation and iterative optimization were used. MSE was used as the loss function, and the Adam optimizer was applied. After 200 training epochs, the minimum losses achieved were 0.0038 for the LSTM model, 0.0020 for the CNN-LSTM model, and 0.0156 for the RF model (Figure 2).

### Evaluation of Predictive Performance

The overall performance of the 4 time-series forecasting models in predicting ILI counts is summarized in the table. The results demonstrate that the deep learning models substantially outperformed the traditional statistical model. Among them, the CNN-LSTM hybrid model achieved the best performance, with an *R*² of 0.7623, RMSE of 0.3433, MAE of 0.1623, and MAPE of 9.2142%. The RF model exhibited comparable accuracy to CNN-LSTM, yielding an *R*² of 0.7624 and RMSE of 0.3433, though its MAPE (10.6332%) was slightly higher. In contrast, the SARIMA model performed poorest, with an *R*² of –0.2645, RMSE as high as 0.8863, MAE of 0.7837, and MAPE of 62.7447% (Table 1).

**Table 1.** Performance comparison of univariate time-series forecasting models.

Model	R^2^^a^	RMSE^b^	MAE^c^	MAPE^d^ (%)
LSTM^e^	0.7287	0.3668	0.1697	9.6114
CNN-LSTM^f^	0.7623	0.3433	0.1623	9.2142
RF^g^	0.7624	0.3433	0.1921	10.6332
SARIMA^h^	−0.2645	0.8863	0.7837	62.7447

a R2: coefficient of determination.

b RMSE: root-mean-squared error.

c MAE: mean absolute error.

d MAPE: mean absolute percentage error.

e LSTM: long short-term memory.

f CNN-LSTM: convolutional neural network–long short-term memory.

g RF: random forest.

h SARIMA: Seasonal Autoregressive Integrated Moving Average.

### Model Performance Across Different Forecast Horizons

To assess the stability and accuracy of the models over varying prediction periods, performance was compared across three forecast horizons: 8 weeks, 12 weeks, and 26 weeks (Table 2). For short-term forecasting (8 wks), all deep learning models performed well. CNN-LSTM (*R*²=0.87, RMSE=0.07, MAE=0.06, and MAPE=5.61%), LSTM (*R*²=0.87, RMSE=0.07, MAE=0.06, and MAPE=5.47%), and RF (*R*²=0.88, RMSE=0.07, MAE=0.06, and MAPE=5.83%) achieved similar accuracy, with all error metrics remaining at low levels. When the forecast horizon was extended to 12 weeks, model performance declined slightly but remained stable: CNN-LSTM (*R*²=0.86), LSTM (*R*²=0.86), and RF (*R*²=0.87) continued to perform closely, with MAPE values ranging between 6% and 6.3%. In long-term forecasting (26 wks), the CNN-LSTM model demonstrated stronger robustness, attaining an *R*² of 0.89, RMSE of 0.11, MAE of 0.08, and MAPE of 7.03%, which was notably better than LSTM (*R*²=0.91, but MAPE=6.86%) and RF (*R*²=0.91 and MAPE=7.29%). The SARIMA model performed poorly across all forecast horizons. For the 8-week horizon, it produced an *R*² of –14.82 and an MAPE as high as 76.58%; in the 26-week horizon, its performance improved slightly (*R*²=–0.56 and MAPE=85.12%), yet remained substantially inferior to the deep learning models (Table 2).

**Table 2.** Performance of univariate models across different forecasting horizons.

Model	Scale (week)	R^2^^a^	RMSE^b^	MAE^c^	MAPE^d^ (%)
RF^e^	8	0.88	0.07	0.06	5.83
RF	12	0.87	0.07	0.06	6.3
RF	26	0.91	0.1	0.08	7.29
LSTM^f^	8	0.87	0.07	0.06	5.47
LSTM	12	0.86	0.08	0.06	6.15
LSTM	26	0.91	0.1	0.08	6.86
CNN-LSTM^g^	8	0.87	0.07	0.06	5.61
CNN-LSTM	12	0.86	0.07	0.06	6.17
CNN-LSTM	26	0.89	0.11	0.08	7.03
SARIMA^h^	8	−14.82	0.81	0.76	76.58
SARIMA	12	−6.88	0.91	0.82	85.12
SARIMA	26	−0.56	0.88	0.8	72.65

a R2: coefficient of determination.

b RMSE: root-mean-squared error.

c MAE: mean absolute error.

d MAPE: mean absolute percentage error.

e RF: random forest.

f LSTM: long short-term memory.

g CNN-LSTM: convolutional neural network–long short-term memory.

h SARIMA: Seasonal Autoregressive Integrated Moving Average.

### ILI Prediction Incorporating Multisource Data

#### Model Construction and Hyperparameter Settings

As the integration of multisource data expanded the input dimensionality from 1 to 6, the model complexity increased significantly. To strike a balance between parameter search efficiency and predictive performance, a progressive hyperparameter tuning strategy based on the univariate model configurations was adopted. Specifically, using the optimal parameters of the univariate models as a baseline, we iteratively fine-tuned the network depth, number of hidden units, and learning rate based on the validation set performance. The final optimal configurations were determined as follows: the LSTM model comprises 2 LSTM layers with 128 and 64 hidden units, a batch size of 13, and a learning rate of 0.0001. The CNN-LSTM hybrid model uses 3 CNN channels (configured as [1,32], [1,64], and [1,128]) followed by 2 LSTM layers (with 128 and 64 hidden units), a batch size of 32, and a learning rate of 0.001. For the RF model, the number of trees is set to 200, the maximum tree depth to 4, the minimum number of samples required to split an internal node to 2, and the minimum number of samples required to be at a leaf node to 2. For the Transformer model, the input dimension varies between 2 and 6 depending on the specific predictor combination. The model is configured with an attention hidden dimension of 512, 4 encoder layers, 4 attention heads, an output dimension of 1, a sliding window size of 6, a batch size of 13, and a learning rate of 0.0003 across 200 training epochs. Notably, during the forward pass of the Transformer, the input features are first upsampled from their initial dimension to 32 via a 1-dimensional convolutional layer, then processed by the Transformer encoder, and finally mapped to the prediction output through an adaptive average pooling layer and a linear layer.

#### Performance Evaluation of Different Factor Combinations

##### Overview

After model construction, retrospective forecasts were generated using the 4 models and compared against the test set. Building on the model architectures and hyperparameter configurations described above, the predictive performance of the LSTM, CNN-LSTM, Transformer, and RF models under different feature combinations was systematically evaluated (Table 3).

**Table 3.** Predictive performance of multisource data models under different feature combinations (evaluated by *R*²^a^ and MAPE)^b^.

Model and features	*R* ^2^ ^a^	MAPE^b^
Transformer		
ILI^c^ + Meteorology	0.4050	206.3577
ILI + BMS^d^	0.4175	124.9878
ILI + SBI^e^	0.5593	78.4199
ILI + Meteorology + BMS	0.4834	88.3482
ILI + Meteorology + SBI	0.3739	230.3408
ILI + BMS + SBI	0.0961	306.6268
ILI + Meteorology + BMS + SBI	0.5301	93.6046
ILI + Meteorology + BMS + SBI + SI^f^	0.7007	22.7379
CNN-LSTM^g^		
ILI + Meteorology	0.6767	119.7572
ILI + BMS	0.6671	115.5503
ILI + SBI	0.8113	54.1171
ILI + Meteorology + BMS	0.7939	146.3055
ILI + Meteorology + SBI	0.6704	203.4079
ILI + BMS + SBI	0.6726	176.558
ILI + Meteorology + BMS + SBI	0.6176	138.4909
ILI + Meteorology + BMS + SBI + SI	0.5392	27.9462
LSTM^h^		
ILI + Meteorology	0.7982	56.4777
ILI + BMS	0.7625	82.3763
ILI + SBI	0.7681	56.6477
ILI + Meteorology + BMS	0.7395	76.8989
ILI + Meteorology + SBI	0.8101	51.7079
ILI + BMS + SBI	0.7552	82.2306
ILI + Meteorology + BMS + SBI	0.8350	63.7849
ILI + Meteorology + BMS + SBI + SI	0.7124	9.6521
RF^i^		
ILI + Meteorology	-0.4361	34.0147
ILI + BMS	0.1001	29.7619
ILI + SBI	0.0764	39.4656
ILI + Meteorology + BMS	0.0603	27.8783
ILI + Meteorology + SBI	0.0402	37.6212
ILI + BMS + SBI	0.6067	24.2605
ILI + Meteorology + BMS + SBI	0.6700	24.0408
ILI + Meteorology + BMS + SBI + SI	0.6186	23.89

a R2: coefficient of determination.

b MAPE: mean absolute percentage error.

c ILI: influenza-like illness.

d BMS: Baidu Migration Scale.

e SBI: synthetic Baidu index.

f SI: Stringency Index.

g CNN-LSTM: convolutional neural network–long short-term memory.

h LSTM: long short-term memory.

i RF: random forest.

##### Transformer Model

The Transformer model exhibited considerable variation in performance across different feature sets. When only historical ILI data and meteorological factors were used, the model showed a low fit (*R*²=0.405) and large prediction error (MAPE=206.36%). After progressively introducing additional features, the synthetic Baidu index (SBI) proved most beneficial among single external features: the combination of ILI + SBI achieved an *R*² of 0.5593 and an MAPE of 78.42%. Among 2-factor combinations, the ILI +  meteorology +BMS Index set performed relatively well (*R*²=0.4834 and MAPE=88.35%). For multifactor combinations, the ILI +  meteorology + BMS +  SBI combination yielded improved results, with *R*² rising to 0.5301 and MAPE decreasing to 93.60%. In the full-feature scenario, the inclusion of the Oxford SI led to a marked performance gain. Under the complete feature set (ILI +  meteorology + BMS +  SBI + SI), the Transformer model achieved its best predictive outcome, with *R*² significantly increasing to 0.7007 and MAPE substantially dropping to 22.74%.

##### CNN-LSTM Hybrid Model

For the CNN-LSTM hybrid model, the combination of historical ILI data and meteorological factors alone provided a relatively good fit (*R*²=0.6767), although prediction error remained high (MAPE=119.76%). The BMS index performed similarly to meteorological factors (*R*²=0.6671 and MAPE=115.55%). In contrast, the BSI again showed a pronounced advantage: the ILI +  SBI combination achieved an *R*² of 0.8113 and an MAPE of 54.12%, substantially outperforming other single-factor combinations. Among 2-factor combinations, the ILI +  meteorology + BMS set performed relatively well (*R*²=0.7939 and MAPE=146.31%). However, when multiple factors were included, the model’s fit declined: for the ILI +  meteorology + BMS +  SBI combination, *R*² decreased to 0.6176 and MAPE increased to 138.49%. In the full-feature combination (ILI +  meteorology + BMS +  SBI + SI), the inclusion of the Oxford SI led to a further reduction in *R*² (to 0.5392), but MAPE dropped markedly to 27.95%, indicating that the model retained a strong ability to control relative error under the complete set of predictors.

##### LSTM Model

The LSTM model achieved consistently high fitting performance across all feature combinations, with *R*² values exceeding 0.7 in every scenario. Among single external-feature additions, all combinations attained *R*²>0.76. The ILI +  meteorology combination performed best (*R*²=0.7982 and MAPE=56.48%), while the ILI +  SBI combination also showed comparable results (*R*²=0.7681 and MAPE=56.65%). In 2-factor combinations, the ILI +  meteorology + SBI set exhibited particularly strong performance, with *R*² rising to 0.8101 and MAPE decreasing to 51.71%, representing the optimal outcome among dual-factor configurations. The best overall performance was observed in the full-factor combination excluding the SI: ILI +  meteorology + BMS +  SBI achieved an *R*² of 0.8350 and an RMSE of 0.4507. Consistent with the pattern observed for the CNN-LSTM model, the introduction of the SI led to a decline in *R*² (to 0.7124), but the model’s absolute error metrics improved substantially: RMSE decreased to 0.2821, MAE decreased to 0.2139, and MAPE dropped markedly to 9.65%. This indicates that the SI feature effectively enhanced the predictive accuracy of the LSTM model in terms of error control.

##### RF Model

The RF model exhibited considerable variability in overall fitting performance across different feature combinations. When only a single external feature was added, the inclusion of meteorological factors alone resulted in a negative *R*² value (*R*²=–0.4361, MAPE=34.01%). Other single-factor combinations also demonstrated generally low predictive performance. Among two-factor combinations, the ILI +  BMS + SBI set performed relatively well, with *R*² increasing to 0.6067 and MAPE reaching 24.26%, representing the best outcome within dual-factor configurations. The optimal combination, excluding the SI, was the full-factor set ILI +  meteorology + BMS +  SBI, which achieved an *R*² of 0.67, RMSE of 0.4128, MAE of 0.3438, and MAPE of 24.04%. After introducing the SI, *R*² decreased slightly to 0.6186, while MAPE dropped to 23.89%, the lowest observed across all scenarios for this model.

A cross-model comparison from the feature perspective revealed notable differences in performance across feature combinations. Regarding the BSI, deep learning models consistently demonstrated effective usage of this feature. In the CNN-LSTM model, the ILI +  SBI combination achieved *R*²=0.8113 and MAPE=54.12%, representing the best single-feature performance for this model. The same combination also yielded stable results in the LSTM model (*R*²=0.7681 and MAPE=56.65%), and was the top-performing single-feature addition for the Transformer model (*R*²=0.5593 and MAPE=78.42%). After introducing the SI, all models exhibited a pronounced reduction in MAPE. The LSTM model showed the most substantial decrease, with MAPE dropping from 63.78% to 9.65% (an 84.87% reduction). Similarly, MAPE for the Transformer model declined from 93.60% to 22.74% (75.70% reduction), and for the CNN-LSTM model from 138.49% to 27.95% (79.82% reduction). The RF model also recorded a slight improvement, with MAPE decreasing from 24.04% to 23.89%. Meteorological factors displayed marked variability across models. While the ILI +  meteorology combination performed well in the LSTM model (*R*²=0.7982 and MAPE=56.48%), it resulted in a negative *R*² value (*R*²=–0.4361) in the RF model. BMS produced the strongest response in the RF model, where the ILI +  BMS + SBI combination reached *R*²=0.6067, substantially outperforming meteorological-only (*R*²=–0.4361) or SBI only (*R*²=0.0764) additions. In the deep learning models, BMS alone yielded moderate performance (LSTM: *R*²=0.7625; Transformer: *R*²=0.4175; CNN-LSTM: *R*²=0.6671), but contributed synergistic effects when combined with other features.

**Figure 3.**
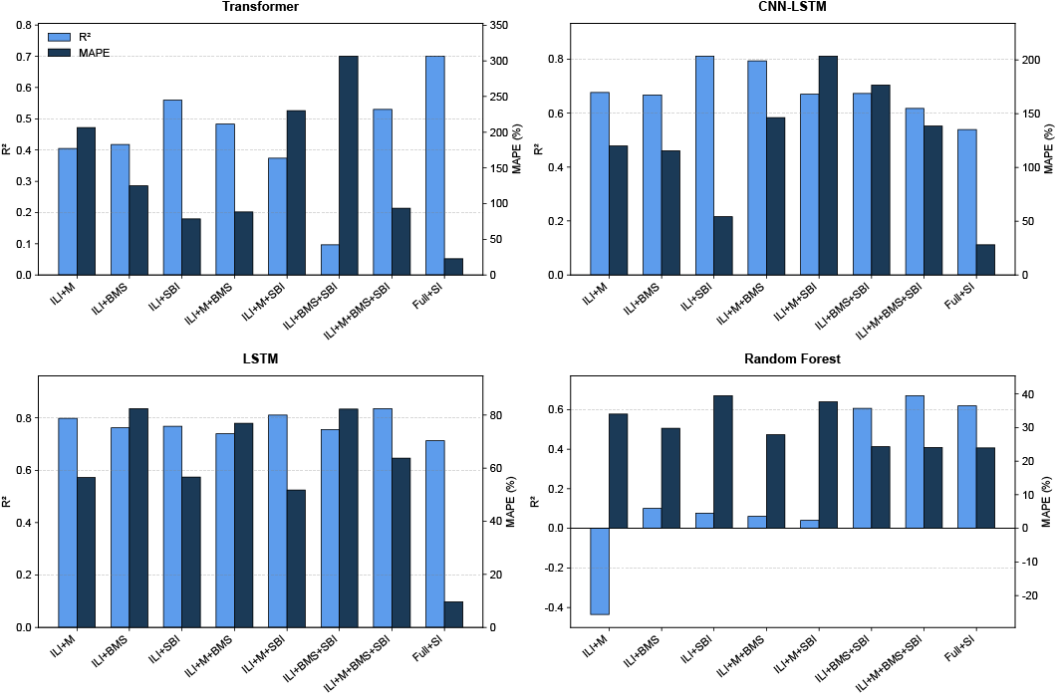
Predictive performance of multisource data models across different feature combinations. BMS: Baidu Migration Scale; CNN-LSTM: convolutional neural network–long short-term memory; ILI: influenza-like illness; LSTM: long short-term memory; M: Meteorology; MAPE: mean absolute percentage error; *R*^2^: coefficient of determination; RF: random forest; SBI: synthetic Baidu index; SI: Stringency Index.

From the perspective of multifeature integration capability, the LSTM model demonstrated the strongest feature fusion performance, maintaining *R*² values above 0.7 across all combinations and achieving the highest fit in the ILI +  meteorology + BMS +  SBI combination (*R*²=0.8350). The Transformer model showed higher sensitivity to feature composition. While some 2-factor combinations (ILI +  BMS + SBI: *R*²=0.0961) led to a sharp decline in performance, the full-feature set yielded the model’s best overall outcome (*R*²=0.7007 and MAPE=22.74%). The CNN-LSTM hybrid model exhibited a decreasing trend in fitting performance as more features were integrated, with *R*² dropping from 0.8113 (ILI +  SBI) to 0.5392 (full-feature set), suggesting certain limitations in its architecture for modeling complex feature interactions. The RF model displayed comparatively weaker overall fitting capability; however, its MAPE remained relatively stable across scenarios (23.89%‐39.47%), indicating consistent control over relative prediction error (Figure 3).

##### Performance of Optimal Feature-Model Combinations Across Forecasting Horizons

**Figure 4.**
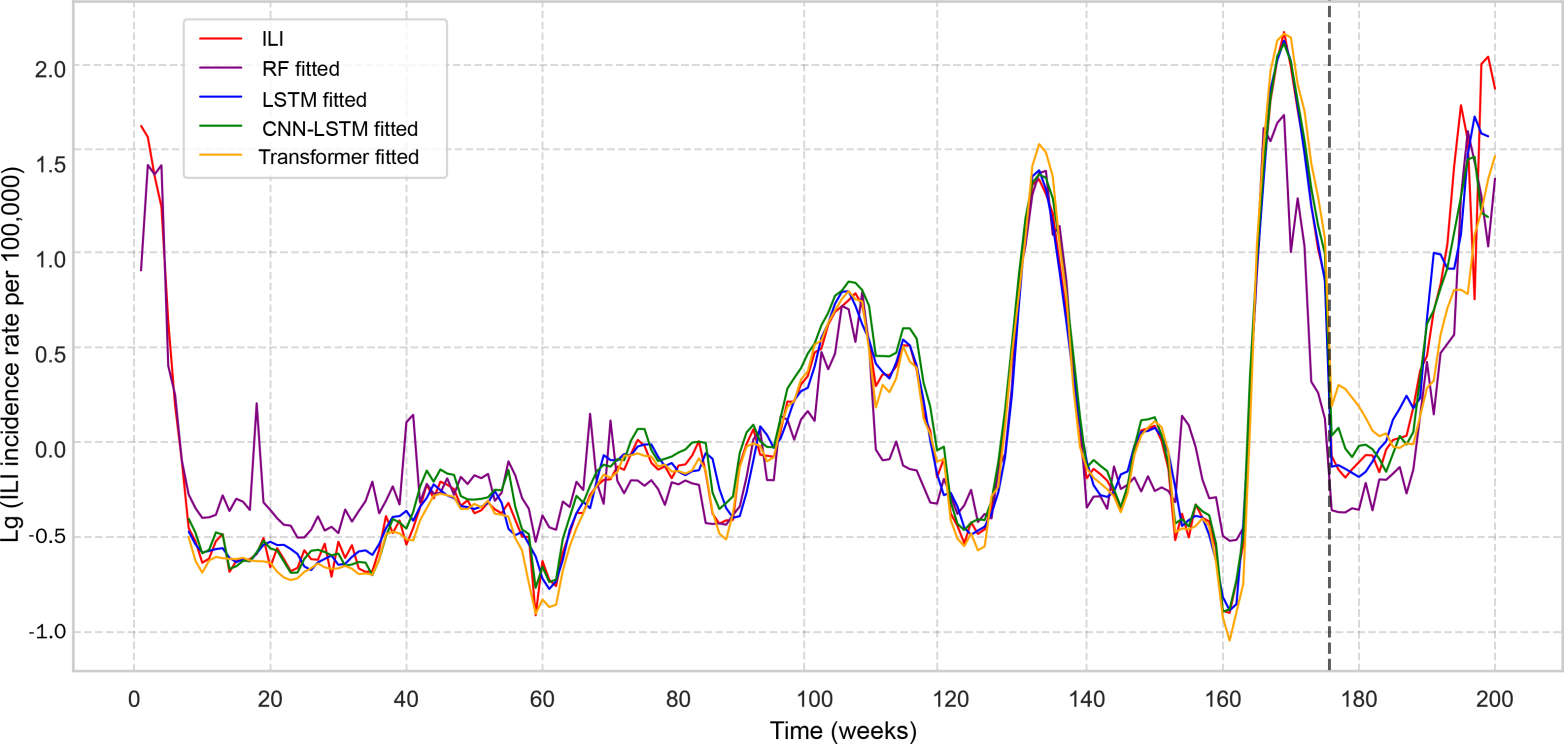
Time-series comparison between fitted values and actual observations from multisource models. CNN-LSTM: convolutional neural network–long short-term memory; ILI: influenza-like illness; LSTM: long short-term memory; RF: random forest.

**Figure 5.**
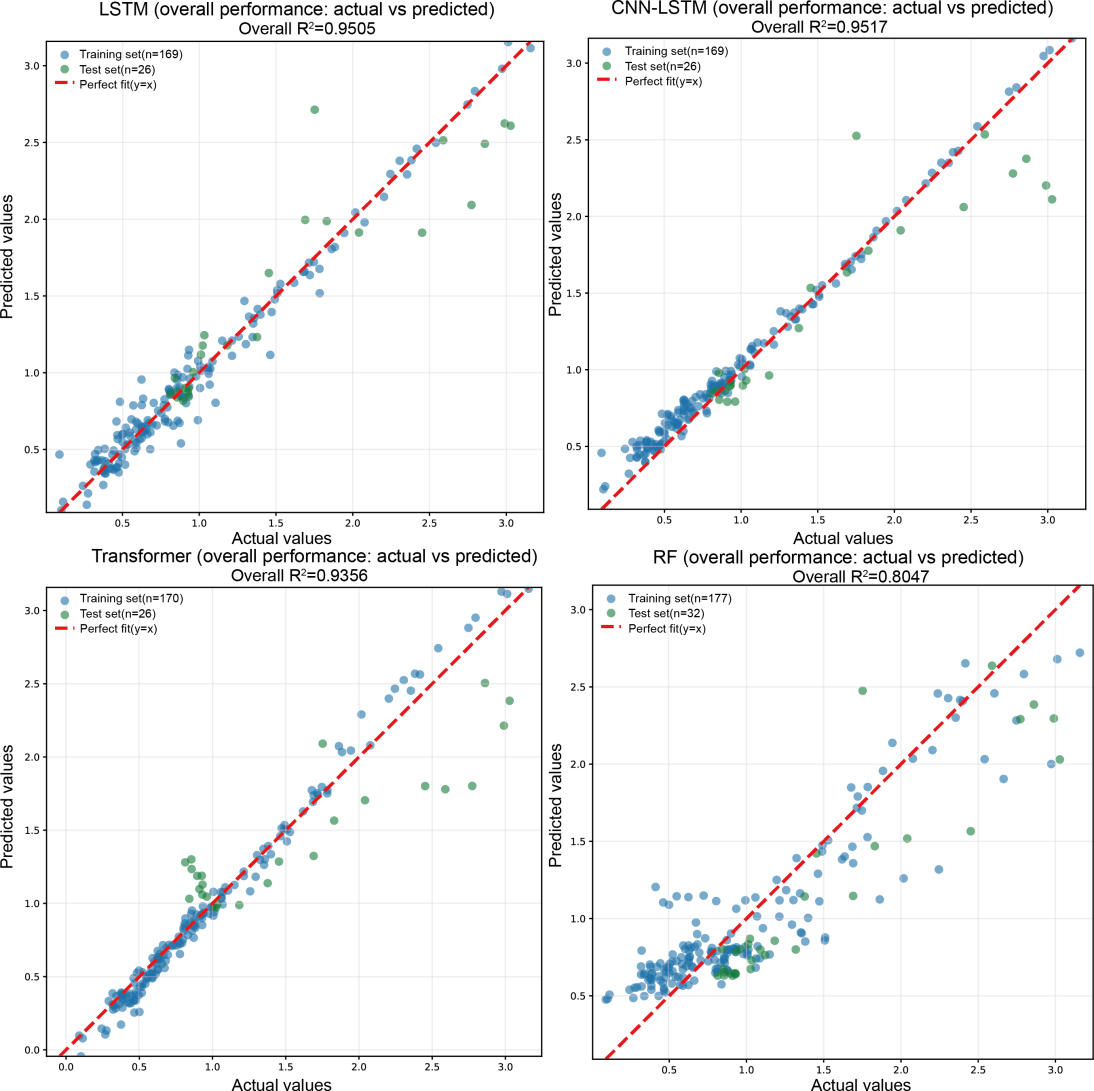
Scatter plots of predicted vs actual values for each model. CNN-LSTM: convolutional neural network–long short-term memory; LSTM: long short-term memory; RF: random forest; *R*^2^: coefficient of determination.

To further examine the robustness of the models across different time scales, the optimal feature combination for each model was selected and evaluated over short-term (8 wks), medium-term (12 wks), and long-term (26 wks) forecasting horizons. Both the LSTM and Transformer models demonstrated relatively stable performance from short- to long-term forecasts. In contrast, the CNN-LSTM and RF models exhibited pronounced variability across different time horizons. The LSTM model showed excellent and consistent fitting ability in short- and medium-term predictions, achieving *R*² values of 0.83 and 0.89, respectively, and maintaining *R*²=0.82 in the 26-week long-term forecast. However, its MAPE remained high across all horizons (63.13%‐85.94%). The Transformer model attained the lowest MAPE (9.69%) together with a high *R*² (0.77) in the 8-week forecast. Although its metrics gradually declined as the horizon extended, it consistently delivered the best MAPE performance throughout all time scales (9.69%‐22.73%). The CNN-LSTM model displayed substantial improvement as the forecast horizon lengthened, with *R*² increasing from 0.26 at 8 weeks to 0.81 at 26 weeks. The RF model exhibited the greatest fluctuation across time scales. It completely failed in the 8-week forecast (*R*²=–4.14) but recovered to *R*²=0.67 in the 26-week horizon. Notably, its MAPE remained at relatively low levels throughout (24.04%‐31.92%; Table 4 and Figures 4 and 5).

**Table 4.** Performance of multisource data models across different forecasting horizons.

Model and scale (week)	R^2^^a^	RMSE^b^	MAE^c^	MAPE^d^ (%)
CNN-LSTM^e^				
8	0.26	0.39	0.24	59.79
12	0.53	0.33	0.19	51.47
26	0.81	0.46	0.29	54.11
LSTM^f^				
8	0.83	0.19	0.14	82.81
12	0.89	0.16	0.12	63.13
26	0.82	0.45	0.30	85.94
Transformer				
8	0.77	0.15	0.13	9.69
12	0.68	0.19	0.16	15.46
26	0.70	0.42	0.35	22.73
RF^g^				
8	−4.14	0.32	0.31	31.92
12	0.27	0.35	0.30	25.72
26	0.67	0.41	0.34	24.04

a R2: coefficient of determination.

b RMSE: root-mean-squared error.

c MAE: mean absolute error.

d MAPE: mean absolute percentage error.

e CNN-LSTM: convolutional neural network–long short-term memory.

f LSTM: long short-term memory.

g RF: random forest.

**Figure 6.**
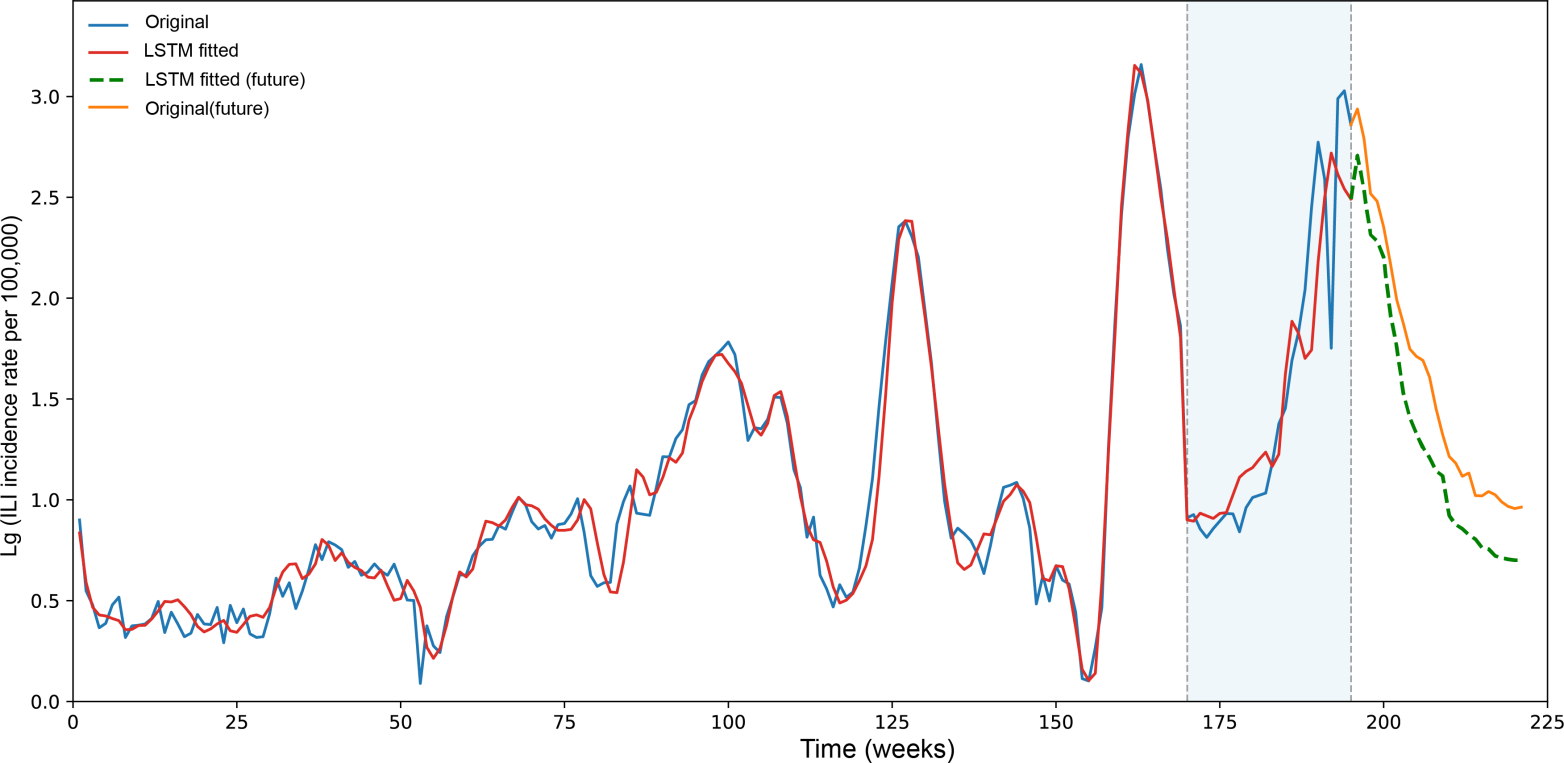
Projected 26-week trend of influenza-like illness incidence in Hubei Province.

Based on the comprehensive performance of the multisource data models, we selected the optimal model and feature combination—the LSTM architecture integrating ILI, meteorological variables, BMS, and SBI—to prospectively forecast the incidence trends of ILI in Hubei Province for weeks 1‐26 of 2024 and to conduct external validation. The predictive results indicate that the ILI incidence in Hubei Province would experience a precipitous decline followed by a plateau, suggesting a gradual resolution of the epidemic and an eventual return to pre-epidemic baseline levels. Notably, this prediction window coincides with the 2024 winter-spring influenza epidemic season. The forecasted trajectory comprised 3 distinct temporal phases. First, during the rapid resolution phase (lasting 6 wks from January to February), the incidence rate exhibits a sharp downward trend. Second, in the slow spring decline phase (lasting approximately 8 wks from March to April), the rate of decrease significantly decelerates. Finally, during the late-spring to early-summer stabilization phase (lasting approximately 9 wks from May to June), the ILI incidence fully reverts to the interepidemic baseline, transitioning the outbreak into a persistent low-prevalence state. The external forecasting results aligned closely with the actual observed incidence trends. Quantitative evaluations yielded an *R*^2^ of 0.7707, an RMSE of 0.2937, an MAE of 0.3854, and a MAPE of 40.73%. Although there was a marginal decrease in predictive efficacy compared to the internal test set (*R*^2^=0.8350), the model maintained a robust overall performance, demonstrating substantial generalizability and reliability for real-world epidemiological surveillance (Figure 6).

## Discussion

### Principal Findings

This study systematically compared the performance of five models—SARIMA, LSTM, CNN-LSTM, Transformer, and RF—in forecasting ILI incidence. The results indicate that deep learning models significantly outperformed the traditional statistical model, and multisource data models exhibited superior predictive accuracy and precision compared with univariate time-series models. In univariate ILI time-series forecasting, CNN-LSTM achieved the best performance, followed by RF and LSTM, while the SARIMA model performed poorest. This finding aligns with the conclusion of Li et al [26], who reported the distinct advantage of deep learning methods in capturing nonlinear patterns in influenza data. Influenza incidence is influenced by multiple factors, including viral mutation, climatic variation, and human behavior, resulting in highly nonlinear and non-stationary characteristics. As noted by Nsoesie et al [32], traditional statistical models have inherent limitations when handling abrupt outbreaks and irregular fluctuations in epidemiological data. In this study, the negative *R*² value obtained by the SARIMA model suggests that its predictions were even less accurate than a simple mean forecast. The SARIMA model relies on assumptions of linearity and stationarity, which limit its ability to adapt to the sudden outbreaks and nonstationary nature of influenza data. In contrast, deep learning models demonstrated clear advantages. The LSTM architecture, through its gating mechanisms, selectively retains and forgets sequential information, effectively capturing long-range temporal dependencies and overcoming the vanishing-gradient problem associated with traditional RNNs [24]. The CNN-LSTM hybrid framework further integrates the local feature-extraction capability of convolutional networks, enabling the recognition of multiscale temporal patterns. Li also reported that CNN-LSTM outperforms single-model architectures in influenza trend prediction [33]. The RF model achieved an *R*² value comparable to that of CNN-LSTM. Its core strength lies in ensemble learning, which reduces variance and enhances prediction stability; under limited sample conditions, ensemble methods remain competitive. Regarding robustness across forecast horizons, model performance followed a predictable pattern as the prediction period extended. In short-term forecasting (8 wks), LSTM, CNN-LSTM, and RF performed similarly, all maintaining high accuracy. When the horizon was extended to 12 and 26 weeks, performance declined slightly but remained generally stable. In contrast, SARIMA performed poorly overall, though its performance improved gradually as the time series lengthened, reflecting its capacity to capture longer-term trends as a classical time-series model.

In multisource data fusion forecasting, this study systematically evaluated the performance of four models: LSTM, CNN-LSTM, Transformer, and RF. The results indicate substantial differences among models in their ability to integrate multiple features. The LSTM model demonstrated the strongest feature-fusion capacity, maintaining *R*² values above 0.71 across all feature combinations and exhibiting the most stable performance. Under the full-feature set (ILI +  meteorology + BMS +  SBI), LSTM achieved the highest fit (*R*²=0.8350 and MAPE=63.78%); after introducing the SI, MAPE decreased sharply to 9.65%. The memory-cell mechanism of LSTM enables it to effectively learn complex temporal relationships among different features, an advantage that is fully realized in multisource data integration scenarios [34]. Specifically, among these integrated variables, the core value of incorporating the SI lies in equipping the models with adaptability to major public health policy shifts. As a dynamic variable, the SI dropped to zero in 2023 following the relaxation of COVID-19 restrictions [20]. However, its continued presence in the feature set implicitly signals the conclusion of policy interventions, enabling the models to effectively distinguish between intervention-influenced baselines (2020‐2022) and natural epidemic baselines (2023 onward). This mechanism proved crucial for maintaining robust forecasting performance in the first full postpandemic influenza season [35]. For instance, integrating the SI into the Transformer model under the full feature set dramatically reduced the MAPE from 93.60% to 22.74%, demonstrating its vital role in correcting post-pandemic baseline shifts. However, the successful integration of such high-dimensional data (including the SI) was not universal across all architectures. The CNN-LSTM hybrid model showed a declining trend in fitting performance as more features were added. It performed best when only SBI was included (*R*²=0.8113), but its performance deteriorated with additional features, dropping to *R*²=0.5392 in the full-feature combination. Notably, CNN-LSTM achieved the best performance in univariate ILI time-series forecasting. This pattern may suggest limitations of the hybrid architecture in modeling high-dimensional feature interactions. Convolutional operations in the CNN layer can introduce information loss when input dimensions increase, potentially impairing the subsequent LSTM layer’s ability to capture temporal dependencies [36]. Zhao et al [36] also noted that CNN-LSTM requires carefully designed network architectures and feature engineering when handling high-dimensional multivariate time series [37]. The Transformer model exhibited high sensitivity to feature composition. Some 2-factor combinations led to a pronounced performance decline, for example, *R*²=0.0961 for ILI +  BMS + SBI and *R*²=0.3739 for ILI +  meteorology + SBI. Nevertheless, under the complete feature set (ILI +  meteorology + BMS +  SBI + SI), the Transformer achieved relatively balanced overall performance. This behavior is related to the self-attention mechanism of the Transformer. As noted by Vaswani et al [28], the Transformer can directly model dependencies between any positions in a sequence, but it demands sufficient data volume and feature completeness to realize its potential. The RF model displayed comparatively weak overall fitting ability, with most feature combinations yielding *R*² below 0.7. However, its MAPE remained relatively stable across scenarios (23.89%‐39.47%), indicating consistent control over relative prediction error.

Regarding the contribution of individual external features, the SBI produced the most pronounced improvement in predictive performance. In the CNN-LSTM model, the ILI +  SBI combination achieved *R*²=0.8113, representing the best performance across all feature combinations for this model. This combination also yielded stable results in the LSTM model and outperformed other single-feature additions in the Transformer model. These findings are consistent with numerous previous studies indicating that search-engine data can effectively complement influenza activity forecasting [33]. The predictive value of search data stems from its ability to reflect real-time public health concerns and symptom-related information-seeking behavior. As noted by Su et al [31] in a study of influenza surveillance in China, when influenza begins to circulate, patients and their families often search for symptom and treatment information online before seeking medical care, resulting in a significant correlation between the SBI and influenza incidence [37]. The SI substantially enhanced the predictive accuracy of all models. After incorporating SI, MAPE decreased markedly across models. The SI synthesizes the intensity of government responses such as school closures, workplace restrictions, and movement controls. Research by Liu et al [38] reported that influenza activity in China during 2020 was substantially lower than in previous years, closely associated with stringent containment measures. The period covered in this study included the COVID-19 pandemic, during which NPIs not only curbed SARS-CoV-2 transmission but also significantly altered influenza transmission dynamics [39]. Incorporating SI into forecasting models allowed quantification of external intervention effects, thereby significantly improving prediction accuracy. BMS elicited the strongest response in the RF model. Whether added as a single factor, in 2-factor sets, or in multifactor combinations, migration data consistently improved model performance. Studies such as that of Fu and Zhu [40] have demonstrated a strong association between population movement patterns and infectious disease spread. In the deep learning models, BMS alone produced moderate performance, but when combined with other features, it exhibited synergistic effects, enhancing overall predictive efficacy. This suggests that population mobility data can also serve as a valuable auxiliary predictor.

Based on the optimal feature combinations identified for each model, this study further evaluated the performance of multisource models over short-term (8 wks), medium-term (12 wks), and long-term (26 wks) forecasting horizons to examine their robustness and applicability across different time scales. Among the models, the LSTM and Transformer models demonstrated relatively stable performance from short- to long-term forecasts. The LSTM model exhibited excellent fitting ability in short- and medium-term predictions, achieving *R*² values of 0.83 and 0.89 for 8-week and 12-week horizons, respectively, and maintained a high *R*² of 0.82 in the 26-week long-term forecast, indicating good temporal stability. The Transformer model also showed consistent predictive performance across the 3 time scales. In contrast, the performance of the CNN-LSTM and RF models improved substantially as the forecast horizon lengthened. For CNN-LSTM, *R*² increased sharply from 0.26 at 8 weeks to 0.81 at 26 weeks. This pattern may be attributed to the convolutional operations in the CNN layer, which require a sufficiently long sequence to effectively extract local temporal features. The shorter 8-week window may have limited feature learning, whereas the 26-week horizon—approaching half a seasonal cycle—enabled the model to capture more complete seasonal patterns [27]. Li et al [33] also noted that CNN-LSTM achieves optimal performance on periodic time series only when provided with adequately long input sequences. The RF model failed completely in the 8-week forecast (*R*²=–4.14) but recovered to *R*²=0.67 in the 26-week horizon, while its MAPE remained relatively low throughout (24.04%‐31.92%). A plausible explanation is that RF has limited capacity to model lag effects, which may exert a weaker influence in longer-term forecasts, thereby improving performance [29]. These observations suggest that the CNN-LSTM and RF models are better suited for long-term trend prediction.

Overall, deep learning methods demonstrate clear advantages in influenza forecasting. In univariate time-series prediction, the CNN-LSTM model achieved the best performance, effectively capturing the nonlinear and complex temporal dependencies in influenza data and significantly outperforming the traditional SARIMA model. All deep learning models exhibited improved performance when multisource data were integrated. Among them, the LSTM, Transformer, and RF models performed best when environmental factors, population mobility data, and internet-based data were incorporated simultaneously. The LSTM model attained the highest overall performance, with *R*² reaching 0.8350 in the full-feature combination. In contrast, although CNN-LSTM excelled in univariate forecasting, its performance declined as input dimensions expanded to 6 (dropping from *R*²=0.8113 with SBI to *R*²=0.5392 in the full-feature combination). This degradation likely stems from architectural limitations: standard 1D convolutions struggle to capture cross-variable interactions among highly heterogeneous features, and max-pooling may discard fine-grained interdimensional temporal details [41]. Furthermore, the expanded parameter space on our limited training set elevates overfitting risks despite dropout regularization [42]. Consequently, CNN-LSTM appears better suited for low-dimensional scenarios (1‐3 features). The value of multisource data fusion should be considered judiciously. Not all external data sources enhance predictive performance, nor can all models effectively integrate multisource information. The SBI and the Oxford SI contributed most substantially to performance improvement. The former reflects public health information-seeking behavior, while the latter quantifies the intensity of government containment measures. The SBI consistently improved the *R*² of all models, whereas the introduction of the SI led to a marked reduction in MAPE across every model. This suggests that incorporating data sources that reflect external environmental changes is crucial for maintaining prediction accuracy during major public health events or policy interventions.

The projection indicates that ILI incidence in Hubei Province will follow an overall trajectory of “rapid decline—gradual decline—stabilization,” suggesting that the epidemic peak will gradually subside and return to pre-epidemic baseline levels. The forecast period corresponds to the 2024 winter-spring influenza season, which coincides with the tail phase of the winter influenza peak. As temperatures rise and population gatherings decrease, ILI incidence is expected to decline rapidly to interepidemic baseline levels, marking a transition to a low-activity state. In summary, ILI incidence in Hubei Province during the 2024 winter-spring period is projected to exhibit typical seasonal waning characteristics, shifting gradually from the winter epidemic peak to a low-incidence phase by late spring and early summer. This overall trend aligns with the established seasonal pattern of influenza in Hubei Province, which is characterized by high activity in winter and spring and low activity in summer.

### Limitations

First, the study focused exclusively on Hubei Province. While this provided an ideal natural experiment to validate model robustness under complex post–COVID-19 dynamics—spanning from extreme low incidence during the 2020 pandemic to a historical peak in 2023—generalizability to other climatic zones and demographic populations requires further investigation. Second, the limited 209-week ILI surveillance timeframe may restrict the full exploitation of data-intensive models. Third, the 2020‐2023 training data coincided with the pandemic, exhibiting extreme heterogeneity. While this enabled models to robustly learn policy-driven dynamics (external validation *R*²=0.7706), their exposure to typical seasonality was limited. Future applications should use transfer learning with post-pandemic data to recalibrate model weights. Finally, as Baidu’s market share declines and platforms like WeChat and Douyin grow for health information seeking, the future representativeness of Baidu-based indices may diminish. Subsequent studies should incorporate multiplatform internet data to ensure long-term model applicability.

## Supplementary material

10.2196/91844 Multimedia Appendix 1 Additional tables, figures, and methodological descriptions.

## Abbreviations

AIC: Akaike information criterion
BIC: Bayesian information criterion
BMS: Baidu Migration Scale
BSI: Baidu Search Index
CNN: convolutional neural network
CNN-LSTM: convolutional neural network–long short-term memory
ILI: influenza-like illness
LSTM: long short-term memory
MAE: mean absolute error
MAPE: mean absolute percentage error
MSE: mean squared error
NPI: nonpharmaceutical intervention
OxCGRT: Oxford COVID-19 Government Response Tracker
RF: random forest
RMSE: root-mean-squared error
RNN: recurrent neural network
SARIMA: Seasonal Autoregressive Integrated Moving Average
SBI: Synthetic Baidu Index
SI: Stringency Index
