# Supplementary material for: Influenza-Like Illness Forecasting Using Multisource Data: Comparative Deep Learning Study
Source: JMIR Med Inform. 2026 Aug 11;14:e91844. doi: 10.2196/91844 (PMC13460793; doi:10.2196/91844)
Supplement: Supplementary file 1 [file medinform-v14-e91844-s001.docx]

**1. Baidu Search Index**

A systematic literature search was conducted on databases such as PubMed and CNKI to identify studies related to influenza-like illness (ILI)[1]. Keywords cited ≥ 2 times in published articles were summarized, resulting in an initial list of 53 candidate keywords potentially associated with influenza epidemic trends (Table S1). These keywords covered categories including early ILI symptoms (e.g., "fever," "cough"), diagnosis, medications, disease prevention, and treatment. This initial list of 53 keywords underwent a multi-step data quality filtering process. First, 3 keywords that could not be queried on the Baidu Index platform were removed. Second, 8 keywords with incomplete time-series data were excluded. Finally, by comparing search trends with the ILI incidence curve in Hubei Province, 25 keywords whose temporal patterns showed no correspondence with ILI activity were discarded, leaving 17 candidate keywords for subsequent analysis. To select keywords most strongly associated with ILI case counts, cross-correlation analysis was employed to calculate the maximum cross-correlation coefficient between the Baidu Index of the 17 remaining keywords and the ILI incidence series, specifically accounting for a time lag of 0–4 weeks. A maximum cross-correlation coefficient ≥ 0.5 was established as the inclusion criterion[2]. This process ultimately yielded 9 core keywords (with maximum cross-correlation coefficients ranging from 0.54 to 0.82, Table S2).To avoid multicollinearity and to construct a consolidated internet-based surveillance indicator, these 9 keywords were aggregated into a Synthesized Baidu Index (SBI) [3]. Specifically, the maximum cross-correlation coefficient of each keyword with ILI incidence was used as its weight for the synthesis, ensuring that keywords with higher predictive contributions received greater weights. Additionally, a correlation heatmap of the 17 candidate keywords and ILI incidence has been newly incorporated (Figures) to intuitively display the screening results and the effectiveness of the multicollinearity processing. The composite index was calculated using the following formula:

$\text{M}^{\text{W}}\text{(}\text{t}\text{)=}\left. \sum_{\text{i}\text{=1}}^{\text{7}} {\frac{\text{C}_{\text{i}}}{\sum_{\text{j}\text{=1}}^{\text{7}} \text{C}_{\text{j}}}\text{M}}_{\text{i}} \right.$	（1）

In Formula (1),${\text{ }\text{M}}^{\text{W}}\text{(}\text{t}\text{)}$ represents the synthesized Baidu Index, $\text{M}_{\text{i}}$ denotes the Baidu Index of the *i-th* keyword, $\text{C}_{\text{i}}$ is the maximum correlation between the Baidu Index of the *i-th* keyword and influenza cases.

**Table S1 Preliminary Screening Results of Keywords.**

| **Keyword** | **Screening** | **Keyword** | **Screening** |
| --- | --- | --- | --- |
| *Influenza A* | *Match* | Influenza B | Mismatch |
| *Influenza A Symptoms* | *Match* | *Influenza Treatment* | *Match* |
| H1N1 Influenza A | Mismatch | H1N1 Influenza | Mismatch |
| *Influenza A Virus* | *Match* | *Seasonal Influenza* | *Match* |
| H1N1 Influenza A Virus | Discontinuous | Influenza Vaccine | Mismatch |
| Main Symptoms of Influenza | Discontinuous | Is Flu Vaccine Necessary | Not Included |
| Flu Outbreak Not Due to COVID Lowering Immunity | Discontinuous | Quadrivalent Flu Vaccine | Discontinuous |
| *Influenza* | *Match* | Flu Vaccine Price | Discontinuous |
| *Influenza Virus* | *Match* | *Oseltamivir* | *Match* |
| Quadrivalent Flu Vaccine | Mismatch | *Effects and Efficacy of Oseltamivir* | *Match* |
| *What Medicine for Flu* | *Match* | Oseltamivir Phosphate Capsule | Mismatch |
| Flu Symptoms | Mismatch | Effects and Efficacy of Oseltamivir Phosphate Granules | Not Included |
| *Flu Vaccine* | *Match* | Oseltamivir Phosphate | Mismatch |
| *Best Medicine for Flu* | *Match* | Cold and Fever | Mismatch |
| Haemophilus influenzae | Mismatch | What Medicine for Flu | Mismatch |
| Difference Between Flu and Common Cold | Mismatch | Best Medicine for Flu | Mismatch |
| Is Flu Vaccine Necessary | Discontinuous | Acute Upper Respiratory Infection | Mismatch |
| *Flu Prevention* | *Match* | Medicine for Cold and Fever | Mismatch |
| Viral Flu | Discontinuous | H1N1 | Mismatch |
| Limb Weakness | Mismatch | Cold Medicine | Mismatch |
| *Fever* | *Match* | 999 Ganmaoling | Not Included |
| *Cough* | *Match* | *Gankang* | *Match* |
| Pediatric Cold | Mismatch | Kuaike | Mismatch |
| Cough Relief | Mismatch | Banlangen Granules | Mismatch |
| Muscle Soreness | Mismatch | *Antipyretic* | *Match* |
| Nasal Congestion | Mismatch | Body Temperature | Mismatch |
| Pyrexia | Mismatch |  |  |

*The keywords in italics represent those retained after the exclusion and re-filtering of the initially screened keywords.

**Table S2 Analysis of Maximum Cross‑Correlation and Corresponding Time Lags between Selected Keywords and ILI Incidence**

| Keyword | Maximum Cross‑Correlation with ILI | Corresponding Lead |
| --- | --- | --- |
| Influenza virus | 0.58 | 0 |
| Seasonal influenza | 0.21 | 0 |
| Influenza A | 0.54 | 2 |
| Influenza A virus | 0.58 | 0 |
| Influenza | 0.28 | 0 |
| Influenza prevention | 0.67 | 1 |
| Influenza treatment | 0.49 | 0 |
| Oseltamivir | 0.75 | 1 |
| Efficacy and function of oseltamiv | 0.82 | 1 |
| Fever | 0.14 | 1 |
| Gan Kang (a common cold medicine) | 0.1 | 1 |
| Cough | 0.12 | 1 |
| Symptoms of influenza A | 0.6 | 1 |
| What medicine to take for influenza | 0.61 | 2 |
| What is the most effective medicine for influenza | 0.7 | 2 |
| Influenza vaccine | 0.11 | 2 |
| Antipyretic | 0.08 | 1 |

**2.Detailed Predictive Model Formulations**

1. SARIMA Model
The standard formulation of the Seasonal Autoregressive Integrated Moving Average (SARIMA) model is denoted as ARIMA(p, d, q)×(P, D, Q)s. The modeling procedure was conducted as follows: Stationarity and seasonal adjustment: The time series plot was examined to assess trend and seasonality. If trend or seasonal fluctuations were present, seasonal differencing of order D (with period s) was applied first, followed by ordinary differencing of order d if necessary, until the series was stationary. Model identification and order selection: For the differenced stationary series, autocorrelation (ACF) and partial autocorrelation (PACF) plots were examined. The cut‑off or trailing characteristics at lags multiples of the seasonal period s were used to preliminarily identify the seasonal orders (P, Q) and non‑seasonal orders (p, q), yielding a set of candidate models of the form (p, d, q)×(P, D, Q) s[4]. Parameter estimation and model selection: Candidate models were fitted using maximum likelihood estimation, and the optimal model was selected based on the lowest Akaike Information Criterion (AIC) and Bayesian Information Criterion (BIC). Model diagnostics: The Ljung–Box test was performed on the residuals to check for white noise (a p‑value >0.05 indicated adequacy). Model fit was further evaluated using AIC/BIC to ensure the residuals were consistent with a white-noise process.

2. LSTM Model

LSTM stores long-term information through a memory cell (cell state) and employs three "gating mechanisms" to control information updating, forgetting, and output, thereby effectively capturing long-term dependencies in time series [5].

Forget Gate (F): Controls which information from the long-term memory$\text{C}_{\text{t}\text{-1}}$ at the previous time step should be forgotten. The forget gate comprises a $\text{σ}$ neural network layer. It takes the hidden state *h_t-1_* from the previous time step and the current input *X_t_* as inputs, generating an output vector ft with values between 0 and 1 through the sigmoid activation function. This process is implemented by the following equation:

$\text{f}_{\text{t}}\text{=}\text{σ}\text{(}\text{W}_{\text{f}}\text{∙}\left[ \text{h}_{\text{t}\text{-1}}\text{,}\text{x}_{\text{t}} \right]\text{+}\text{b}_{\text{f}}\text{)}$ (2)

where $\text{W}_{\text{f}}$ denotes the weight matrix of the forget gate, $\text{b}_{\text{f}}$ is the bias term, and $\text{σ}$ represents the sigmoid function. When elements in $\text{f}_{\text{t}}$ approach 0, the corresponding information should be forgotten; when approaching 1, the corresponding information should be retained.

Input Gate (I): Determines how much new information should be added to the memory cell. It comprises a $\text{σ}$ neural network layer and a tanh neural network layer. In the input gate, the $\text{σ}$ layer outputs *i_t_*, while the tanh layer generates a new candidate value vector $\tilde{\text{C}_{\text{t}}}$. The memory cell *C_t_* is updated by combining the outputs of the forget gate and input gate, integrating *f_t_*,*i_t_*, and $\tilde{\text{C}_{\text{t}}}$*.*

$$\text{i}_{\text{t}}\text{=}\text{σ}\text{(}\text{W}_{\text{i}}\text{∙}\left[ \text{h}_{\text{t}\text{-1}}\text{,}\text{x}_{\text{t}} \right]\text{+}\text{b}_{\text{i}}\text{)}$$

$\tilde{\text{C}_{\text{t}}}\text{=}\tan\text{h}\text{(}\text{W}_{\text{f}}\text{∙}\left[ \text{h}_{\text{t}\text{-1}}\text{,}\text{x}_{\text{t}} \right]\text{+}\text{b}_{\text{c}}\text{)}$ (3)

$$\text{C}_{\text{t}}\text{=}\text{f}_{\text{t}}\text{∙}\text{C}_{\text{t}\text{-1}}\text{+}\text{i}_{\text{t}}\text{×}\tilde{\text{C}_{\text{t}}}$$

where $\text{i}_{\text{t}}$ represents the output vector of the input gate, $\text{σ}$ denotes the sigmoid function, $\text{W}_{\text{i}}$ is the weight matrix of the input gate, and $\text{b}_{\text{i}}$ and${\text{ }\text{b}}_{\text{c}}$ represent bias vector parameters. The *tanh* function is denoted as tanh, $\text{C}_{\text{t}}$ represents the memory cell state at the current time step, and $\text{C}_{\text{t}\text{-1}}$ represents the memory cell state at the previous time step.

Output Gate (O): Determines which information from the updated cell state $\text{C}_{\text{t}}$ can be output to the hidden layer $\text{h}_{\text{t}}$. The $\text{σ}$ layer filters input information and outputs *O_t_* , integrating the updated cell state *Ct*, the output signal *ht-1* from the previous time step, and the input signal *X_t_* from the current time step to produce the output signal ht for the current time step, thereby completing the update of *h_t_*. This gate comprises a $\text{σ}$ neural network layer and a tanh function. The above process is implemented by the following equations:

$\text{O}_{\text{t}}\text{=}\text{σ}\text{(}\text{W}_{\text{O}}\text{∙}\left[ \text{h}_{\text{t}\text{-1}}\text{,}\text{x}_{\text{t}} \right]\text{+}\text{b}_{\text{O}}\text{)}$ (4)

$$\text{h}_{\text{t}}\text{=}\text{O}_{\text{t}}\text{×}\tan\text{h}\left( \text{C}_{\text{t}} \right)$$

where $\text{O}_{\text{t}}$ represents the output vector of the output gate, $\text{W}_{\text{O}}$ denotes the weight matrix of the output gate, and $\text{b}_{\text{O}}$ represents the bias vector.

3.CNN-LSTM Model

The CNN feature extraction module employs a one-dimensional convolutional neural network (Conv1d) structure[6]. This module consists of multiple convolutional blocks stacked sequentially. In the base model configuration, convolutional blocks utilize 64 convolutional kernels, with each convolutional layer employing a kernel size of 3×1 and appropriate padding (padding=1) to preserve the temporal dimension of features. Following the convolution operation, ReLU activation functions are introduced, which effectively mitigate the vanishing gradient problem through their activation properties, significantly enhancing the model's nonlinear representation capability. A max pooling layer (MaxPool1d) is positioned at the end of each convolutional block, with a pooling kernel size of 2 and stride of 1, which moderately reduces feature dimensionality while preserving key features and enhancing model robustness to translational variations. Through layer-by-layer processing in the CNN module, local spatiotemporal features in the original input data are effectively extracted and transformed into high-dimensional feature representations. These features are subsequently fed into the LSTM temporal modeling module for further analysis. Finally, the output from the LSTM module is passed to a fully connected layer (Linear Layer), which maps the LSTM output features to a single-dimensional predicted value.

4. Transformer Model

The Transformer architecture comprises four main components: an input layer, an Encoder, a Decoder, and an output layer[7]. In the Input Layer, the input data first undergoes standardization to improve model convergence speed and stability. Given that the input feature dimension is set to 6, the model utilizes a Fully Connected Layer to map these features into a 128-dimensional space for subsequent processing. Additionally, to address the Transformer's inherent lack of positional information when processing time series, sinusoidal and cosine positional encoding was employed. This encodes sequential information into the time series data through the element-wise addition of the input vectors and the positional encoding vectors, calculated as follows:

*PE_(pos,2i)_=sin(pos/*$\text{10000}^{\text{2}\text{i}\text{/}\text{d}_{\text{model}}}$*)* (5)

*PE_(pos,2i+1)_=cos(pos/*$\text{10000}^{\text{2}\text{i}\text{/}\text{d}_{\text{model}}}$*)*

where (*pos*) denotes the position in the sequence, (*i*) is the dimension, and (*d_model_*) is the embedding dimension.

(2) Encoder Layer: The model design includes 4 encoder layers and 4 self-attention heads to enhance model representation capability and computational efficiency. Each encoder layer consists of two sub-layers: a self-attention sub-layer and a fully connected feed-forward sub-layer. A Dropout layer is applied after each sub-layer for regularization. The final encoder produces a d-dimensional vector to feed to the decoder. The self-attention mechanism in the encoder layer dynamically weights input nodes, thereby computing correlations between different features. The computation formula is as follows:

*Attention(Q,K,V)=softmax(* $\frac{\text{QK}^{\text{T}}}{\sqrt{\text{d}_{\text{k}}}}\text{ }$*)V* (6)

where (*Q*) is the query matrix, (*K*) is the key matrix, (*V*) is the value matrix, and (*d_k_*) is the dimension of the keys.

(3) Decoder Layer: The decoder layer structure is similar to the encoder layer. However, in addition to the self-attention sub-layer and fully connected feed-forward sub-layer, each decoder layer incorporates a third sub-layer that applies self-attention mechanism over the encoder output. The decoder input is typically the prediction from the last time step of the encoder output, with each layer comprising self-attention mechanism and feed-forward neural network components while simultaneously processing encoder outputs.

(4) Output Layer: The decoder output undergoes linear transformation to convert internal representations into final prediction outputs, with output dimension set to 1. Through the output layer, the final decoder layer output is mapped to the target time series, enabling dynamic updating of predictions for future time series. Additionally, considering that influenza incidence rates are continuous values, this study does not employ activation functions in the output layer, maintaining linear output[7].

5. Random Forest Model

During the detailed construction of the Random Forest model, the algorithm employs a dual randomness mechanism involving data samples and features to effectively prevent overfitting[8]. In the initial phase, multiple different sample subsets are generated through random sampling with replacement from the original training set. Each sample subset is then used to train an independent decision tree. Simultaneously, during the node splitting process of each tree, the algorithm randomly selects a feature subset from all available features and searches for the optimal splitting feature strictly within this subset. Each tree grows according to the principle of impurity reduction: the algorithm utilizes features and thresholds that maximize the reduction in the child node's Mean Squared Error (MSE) as splitting criteria. It continuously optimizes node partitions through recursive splitting until the preset stopping conditions are met. Once all individual decision trees are fully trained, the model performs final predictions through ensemble strategies.

6.Model Construction and Validation

Following the initial construction of time series prediction models, comprehensive validation and parameter determination were conducted. For the SARIMA model, initial parameter ranges were identified through the analysis of autocorrelation function (ACF) and partial autocorrelation function (PACF) plots of the original series and its differenced series. A grid search was then employed to identify optimal model configurations. Subsequently, model residuals were subjected to the Box-Ljung test to identify outliers and evaluate distributions. The model with the lowest Akaike Information Criterion (AIC) and Bayesian Information Criterion (BIC) values was selected as the final optimal model.For models including LSTM, CNN-LSTM, Transformer, and Random Forest, performance is heavily influenced by hyperparameters. Considering computational costs and the need to maximize data utilization to provide reliable performance estimates, 5-fold cross-validation was selected after repeated testing. Grid search methods were applied to iterate through various hyperparameter combinations, training models on each subset and calculating the average Mean Squared Error (MSE) across all five subsets. Given the complexity and computational cost associated with multi-source data models, 5-fold cross-validation was first employed in univariate ILI time series models to obtain optimal baseline hyperparameters. For models incorporating multi-source data, these optimal baseline parameters were then fine-tuned accordingly.Finally, to mitigate overfitting, Dropout mechanisms were introduced in all applicable network structures with the parameter set to 0.2. During training, the Adaptive Moment Estimation (Adam) algorithm was used as the optimizer. The Adam algorithm achieves adaptive learning rate adjustments by comprehensively considering the first-order moment (mean) and second-order moment (uncentered variance) information of gradients, thereby ensuring the stable convergence of the loss function.

Figure S1 Hyperparameter Optimization Results of the LSTM Model.

Figure S2 Hyperparameter Optimization Results of the CNN-LSTM Model.

Figure S3 Hyperparameter Optimization Results of the RF Model.

**3. Oxford Stringency Index**

Non-pharmaceutical interventions (NPIs) and policy measures were characterized using the Oxford COVID‑19 Government Response Tracker (OxCGRT), an evaluation framework developed by the Blavatnik School of Government research team to systematically track government policies worldwide during the COVID‑19 pandemic[9]. The OxCGRT provides a longitudinal, cross‑national dataset of government responses from January 1,2020 onward. The project monitors policies and interventions implemented by national and some subnational governments through a series of standardized indicators, enabling comparative analysis of governmental reactions during the evolution of the epidemic. Furthermore, it constructs a set of composite indices to quantify the intensity of these responses. Since January 1, 2020, the dataset has captured 56 variables across 185 national and 210 subnational jurisdictions, covering policy domains such as closures and containment, health, and economic measures. Specific standardized indicators include school closures, suspension of public transport, bans on public gatherings, and other government actions, totaling 21 policy indicators used to assess national pandemic‑control policies. These comprise eight containment-and closure-related indicators, four economic-policy indicators, eight health‑system indicators, and one miscellaneous measure *(*Table S3*).* Each specific measure is scored ordinally based on the degree of enforcement. For example, for school closures: a score of 0 indicates no measures, 1 represents recommended closures, 2 indicates partial closures, and 3 corresponds to full closures. To enable quantitative analysis of government response intensity, the OxCGRT derives four core policy indices. Among these, the Stringency Index (SI) is designed specifically to evaluate the strength of containment policies implemented by governments. The SI integrates eight containment indicators (C1–C8) and one public‑information campaign indicator (H1), specifically: school closures (C1), workplace closures (C2), cancellation of public events (C3), restrictions on gathering size (C4), public‑transport closures (C5), stay‑at‑home requirements (C6), restrictions on internal movement (C7), international travel controls (C8), and public‑information campaigns (H1). The SI is calculated using a standardized weighting approach, taking the simple average of the nine sub‑indices. Raw scores for each indicator are rescaled to a common 0–100 scale, where a higher value reflects stronger implementation of containment measures and stricter restrictions. The calculation proceeds as follows:

First, each categorical indicator is weighted to obtain a sub‑index $\text{I}_{\text{j}\text{,}\text{t}}$ using the following formula:

$$\text{I}_{\text{j}\text{,}\text{t}}\text{=}\left. \text{100×}\frac{\text{v}_{\text{j}\text{,}\text{t}}\text{-0.5×(}\text{F}_{\text{j}}\text{-}\text{f}_{\text{j}\text{,}\text{t}}\text{)}}{\text{N}_{\text{j}}} \right.$$

Where $\text{v}_{\text{j}\text{,}\text{t}}$ is the value of the *j-th* indicator on day *t*,$\text{N}_{\text{j}}$is the maximum possible value of the indicator, $\text{F}_{\text{j}}$ indicates whether the indicator has a flag variable (1 if yes, 0 if no), $\text{f}_{\text{j}\text{,}\text{t}}$ is the actual flag value (0 or 1).The flag variable serves as a supplement to the main policy indicator, specifying whether the policy applies to the entire jurisdiction or only to specific areas. When Flag=0, the policy is targeted at one or more specific regions within the jurisdiction (country, state, or city) rather than the whole territory; when Flag=1, the policy applies uniformly across the entire jurisdiction. Subsequently, the Stringency Index is computed from the relevant sub‑indices${\text{ }\text{I}}_{\text{j}\text{,}\text{t}\text{ }}$as follows:

$$\text{index}\text{=}\frac{\text{1}}{\text{k}}\left. \sum_{\text{j}\text{=1}}^{\text{k}} \text{I}_{\text{J}} \right.$$

Where *k* represents the total number of indicators included in the composite index (for the SI, *k*=9), $\text{I}_{\text{J}}$ denotes the sub‑index score for each indicator, which reflects its stringency level and scope of application (adjusted via the flag variable).

Table S3 OXCGRT Index (Hubei Province)

| **Code** | **Category** | **Code** | **Individual Indicator** | **Score** |
| --- | --- | --- | --- | --- |
| C | Containment and Closure | C1 | School Closing | 3 (0,1,2,3) |
|  |  | C2 | Workplace Closing | 3 (0,1,2,3) |
|  |  | C3 | Cancel Public Events | 2 (0,1,2) |
|  |  | C4 | Restrictions on Gathering Size | 4 (0,1,2,3,4) |
|  |  | C5 | Close Public Transport | 2 (0,1,2) |
|  |  | C6 | Stay-at-Home Requirements | 3 (0,1,2,3) |
|  |  | C7 | Restrictions on Internal Movement | 2 (0,1,2) |
|  |  | C8 | International Travel Controls | 4 (0,1,2,3,4) |
| E | Economic | E1 | Income Support | 2 (0,1,2) |
|  |  | E2 | Debt/Contract Relief for Households | 2 (0,1,2) |
|  |  | E3 | Fiscal Measures | 0 |
|  |  | E4 | Giving International Support | 0 |
| H | Health System | H1 | Public Information Campaign | 2 (0,1,2) |
|  |  | H2 | Testing Policy | 3 (0,1,2,3) |
|  |  | H3 | Contact Tracing | 2 (0,1,2) |
|  |  | H4 | Emergency Investment in Healthcare | 4 (0,1,2,3,4) |
|  |  | H5 | Investment in COVID-19 Vaccines | 5 (0,1,2,3,4,5) |
|  |  | H6 | Facial Coverings | 3 (0,1,2,3) |
|  |  | H7 | Vaccination Policy | 5 (0,1,2,3,4,5) |
|  |  | H8 | Protection of Elderly People | 3 (0,1,2,3) |
| M | Miscellaneous | M1 | Other Responses | 0 |

**4.Predictor Selection**
3.1 Spearman Correlation Analysis
Spearman rank correlation analysis was employed to examine the associations between ILI incidence and potential predictors, including meteorological and air quality variables, population migration indices, the Baidu search index, and the Oxford Stringency Index. Indicators with an absolute correlation coefficient |r| > 0.3 were defined as correlated variables, and those with |r| > 0.6 were classified as strongly correlated. Variables showing statistically significant correlations with ILI incidence and clear public health relevance were prioritized for retention in subsequent models[10].

The Spearman correlation analysis revealed that ILI incidence was strongly positively correlated with the composite Baidu index (r = 0.76). A moderate negative correlation was observed with the Oxford Stringency Index (r = –0.44). Population immigration and emigration indices showed moderate positive correlations with ILI incidence (r = 0.36 and 0.31, respectively). Among meteorological factors, relative humidity and dew point exhibited weak positive correlations with incidence (r = 0.18), while mean temperature showed only a very weak association (r = 0.08) *(*Figure S2*).*

Figure S4 Spearman correlation analysis between predictors and ILI.

3.2 Generalized Additive Model

Given the potential nonlinear relationships between predictors and ILI incidence, a Generalized Additive Model (GAM) was employed to model the exposure–response associations. Compared with conventional parametric models, GAM introduces non‑parametric smooth functions that can flexibly capture nonlinear effects of continuous explanatory variables, while retaining parametric terms for categorical variables, thereby achieving an optimal balance between model assumptions and structural adaptability. Considering the count‑data nature of disease incidence and the presence of over‑dispersion, the model was constructed using a quasi‑Poisson distribution for the response variable. An identity link function was specified to directly estimate the absolute change in incidence rate. The model formulation is as follows:

g$\left( \text{μ} \right)\text{=}\text{α}\text{+}\text{s}_{\text{1}}\left( \text{X}_{\text{1}}\text{,}\text{k} \right)\text{+}\text{s}_{\text{2}}\left( \text{X}_{\text{2}}\text{,}\text{k} \right)\text{+...+}\text{s}_{\text{p}}\left( \text{X}_{\text{p}}\text{,}\text{k} \right)\text{, ...}$

$$\text{μ}\text{=}\text{E}\left( \text{Y} | \text{X}_{\text{1}}\text{,}\text{X}_{\text{2}}\text{ ,...}\text{X}_{\text{p}} \right)$$

where$\text{X}_{\text{i}}$ denote the explanatory variables, *Y* is the response variable, $\text{μ}$ represents the expected value of the response variable, *g*$\left( \text{∙} \right)$ is the link function, $\text{s}_{\text{i}}$ are smooth functions, $\text{α}$ is the intercept, and *k=df-1*,In this study, *Y* corresponds to the reported ILI case count, *X* represents the various associated influencing factors, and *k*=7.

The GAM analysis identified six factors that were statistically significantly associated with ILI incidence (p < 0.05): relative humidity (F = 3.0, p = 0.003), mean dew point (F = 3.0, p = 0.003), the Baidu immigration index (F = 22.3, p < 0.001), the Baidu emigration index (F = 2.6, p = 0.008), the Baidu search index (F = 79.2, p < 0.001), and the Oxford Stringency Index (F = 6.5, p < 0.001). In contrast, mean temperature (F = 1.5, p = 0.17) and the air quality index (F = 0.6, p = 0.75) showed no significant association with ILI incidence (Figure S3)*.* The exposure–response relationships revealed the following patterns. ILI risk exhibited an inverted “N”-shaped relationship with relative humidity, with elevated risk at both very low and very high humidity levels. The relative risk peaked at approximately 80% humidity before gradually declining and stabilising. A similar pattern was observed for mean dew point, where risk initially increased, then decreased, and finally stabilised. Among the environmental factors, mean temperature and air quality index did not show significant associations with ILI incidence. For the social factors, the Baidu search index displayed a nearly linear positive trend, indicating that higher search volumes corresponded to higher ILI risk. Both the Baidu immigration index and the Baidu emigration index showed an initial increase in risk followed by a plateau. The Oxford Stringency Index demonstrated a “W”-shaped fluctuating pattern, with risk initially decreasing, then increasing, and subsequently decreasing again.

**Figure S5** Association between predictors and ILI incidence: GAM analysis.

3.3 Inclusion of Predictors
Based on the correlation analysis and the statistically significant associations identified in the GAM analysis, variables that showed significant relationships with ILI incidence were retained for model construction. The final set of predictors included mean temperature, relative humidity, mean dew point, the composite Baidu index, the immigration index, the emigration index, and the Oxford Stringency Index. These variables were used to build a multivariate time‑series forecasting framework. The raw dataset comprised 209 weeks of influenza‑related surveillance data. The six feature variables incorporated were: relative humidity, temperature, mean dew point, Baidu immigration index, Baidu emigration index, and Baidu search index.

**Figure S6**. Time series of ILI incidence and lg ILI incidence in Hubei Province, 2020-2023.

**Figure S7**. Time series plot of the first‑differenced series.

**Figure S8**. ACF and PACF plots of the first‑differenced series.

**Figure S9**. Parameter Grid Search: AIC Values for SARIMA Specifications.

**Figure S10**. Parameter Grid Search: BIC Values for SARIMA Specifications.

**Figure S11**. Seasonal decomposition of the original ILI incidence time series in Hubei Province, 2020‑2023.

**Figure S12**. Feature Importance Analysis Based on SHAP Values.

Table S4. Absolute prediction errors of multi-source data models under different feature combinations (evaluated by RMSE and MAE).

|  | RMSE | MAE |
| --- | --- | --- |
| Transformer |  |  |
| ILI+Meteorology | 0.8247 | 0.7182 |
| ILI+BMS | 0.8159 | 0.5848 |
| ILI+SBI | 0.7097 | 0.4689 |
| ILI+Meteorology+BMS | 0.7684 | 0.5424 |
| ILI+Meteorology+SBI | 0.8459 | 0.7258 |
| ILI+BMS+SBI | 1.1190 | 0.9258 |
| ILI+Meteorology+BMS+SBI | 0.7329 | 0.5306 |
| ILI+Meteorology+BMS+SBI+SI | 0.4192 | 0.3469 |
| CNN-LSTM |  |  |
| ILI+meteorology | 0.6078 | 0.4837 |
| ILI+BMS | 0.6169 | 0.4685 |
| ILI+SBI | 0.4643 | 0.2931 |
| ILI+Meteorology+BMS | 0.4853 | 0.4930 |
| ILI+Meteorology+SBI | 0.6138 | 0.5108 |
| ILI+BMS+SBI | 0.6117 | 0.5282 |
| ILI+Meteorology+BMS+SBI | 0.6613 | 0.5478 |
| ILI+Meteorology+BMS+SBI+SI | 0.5202 | 0.4010 |
| LSTM |  |  |
| ILI+Meteorology | 0.4803 | 0.2985 |
| ILI+BMS | 0.5211 | 0.3499 |
| ILI+ SBI | 0.5148 | 0.3121 |
| ILI+Meteorology+BMS | 0.5456 | 0.3686 |
| ILI+Meteorology+ SBI | 0.4659 | 0.2883 |
| ILI+BMS+ SBI | 0.6289 | 0.3805 |
| ILI+Meteorology+BMS+ SBI | 0.4507 | 0.3323 |
| ILI+Meteorology+BMS+ SBI+SI | 0.2821 | 0.2139 |
| RF |  |  |
| ILI+ Meteorology | 0.8570 | 0.6032 |
| ILI+BMS | 0.6784 | 0.4712 |
| ILI+SBI | 0.6873 | 0.5798 |
| ILI+ Meteorology+BMS | 0.6933 | 0.4712 |
| ILI+ Meteorology+SBI | 0.7294 | 0.5929 |
| ILI+BMS+SBI | 0.4485 | 0.3583 |
| ILI+ Meteorology+BMS+SBI | 0.4128 | 0.3438 |
| ILI+ Meteorology+BMS+SBI+SI | 0.4417 | 0.3538 |

**References**

1.	Zhang Y, Yakob L, Bonsall MB, Hu W. Predicting seasonal influenza epidemics using cross-hemisphere influenza surveillance data and local internet query data. Scientific Reports. 2019 2019-03-01;9(1):3262. doi: 10.1038/s41598-019-39871-2.

2.	Ginsberg J, Mohebbi MH, Patel RS, Brammer L, Smolinski MS, Brilliant L. Detecting influenza epidemics using search engine query data. Nature. 2009 2009-02-19;457(7232):1012-4. doi: 10.1038/nature07634.

3.	Dai S, Han L. Influenza surveillance with Baidu index and attention-based long short-term memory model. PloS One. 2023 2023;18(1):e0280834. doi: 10.1371/journal.pone.0280834.

4.	Kendall MG, Box GEP, Jenkins GM. Time Series Analysis, Forecasting and Control. Journal of the Royal Statistical Society Series A (General). 1971 1971;134(3):450. doi: 10.2307/2344246.

5.	Hochreiter S, Schmidhuber J. Long Short-Term Memory. Neural Computation. 1997 1997/11/1;9(8):1735-80. doi: 10.1162/neco.1997.9.8.1735.

6.	Li G, Li Y, Han G, Jiang C, Geng M, Guo N, et al. Forecasting and analyzing influenza activity in Hebei Province, China, using a CNN-LSTM hybrid model. BMC public health. 2024 2024-08-12;24(1):2171. doi: 10.1186/s12889-024-19590-8.

7.	Vaswani A, Shazeer N, Parmar N, Uszkoreit J, Jones L, Gomez AN, et al. Attention Is All You Need. 2023 2023-08-02. doi: 10.48550/arXiv.1706.03762.

8.	Breiman L. Random forests. Machine learning. 2001 2001/1/1.

9.	Hale T, Angrist N, Goldszmidt R, Kira B, Petherick A, Phillips T, et al. A global panel database of pandemic policies (Oxford COVID-19 Government Response Tracker). Nature Human Behaviour. 2021 2021-04;5(4):529-38. doi: 10.1038/s41562-021-01079-8.

10.	Wang D, Lei H, Wang D, Shu Y, Xiao S. Association between Temperature and Influenza Activity across Different Regions of China during 2010-2017. Viruses. 2023 2023-02-21;15(3):594. doi: 10.3390/v15030594.
